# Bulk ferroelectric heterostructures

**DOI:** 10.1126/sciadv.aef9861

**Published:** 2026-10-02

**Authors:** Yizhe Li, Ziqi Yang, Ying Chen, Zhenbo Zhang, Yun-Long Tang, Matthew Smith, Matthew Lindley, Xuezhen Cao, Annette K. Kleppe, Egor Koemets, David G. Hopkinson, Andrew J. Bell, Steven J. Milne, Antonio Feteira, Sarah J. Haigh, Alexander S. Eggeman, Juncheng Pan, Jiajun Shi, Yuge Yang, David A. Hall

**Affiliations:** ^1^ Department of Materials, University of Manchester, M13 9PL Manchester, UK.; ^2^ Henry Royce Institute, University of Manchester, M13 9PL Manchester, UK.; ^3^ Center for Adaptive System Engineering, School of Creativity and Arts, ShanghaiTech University, Shanghai 201210, China.; ^4^ Shenyang National Laboratory for Materials Science, Institute of Metal Research, Chinese Academy of Sciences, Wenhua Road 72, Shenyang 110016, China.; ^5^ Diamond Light Source Ltd., Harwell Science and Innovation Campus, OX11 0DE Didcot, UK.; ^6^ electron Physical Science Imaging Centre, Diamond Light Source Ltd., Harwell Science & Innovation Campus, OX11 0DE Didcot, UK.; ^7^ School of Chemical and Process Engineering, University of Leeds, LS2 9JT Leeds, UK.; ^8^ Materials and Engineering Research Institute, Sheffield Hallam University, S1 1WB Sheffield, UK.

## Abstract

Interfaces in oxide heterostructures act as critical regions to induce emergent and novel types of functionality in thin films. Herein, we demonstrate the creation of bulk ferroelectric heterostructures (BFHs) in monolithic ferroelectrics by nanoscale elemental partitioning, unlocking functionalities beyond the reach of conventional ferroelectrics and thin-film devices. The unique percolating, compositionally modulated networks act as built-in heterointerfaces that generate coupled ferroelectric-electrostatic-elastic fields, which stabilize charged domain walls and imprint predefined domain configurations throughout the bulk. In exemplar BiFeO_3_-BaTiO_3_ ceramics, the BFH approach raises the Curie temperature to 824°C (increment Δ*T*_C_ > 350°C) and yields superior high-temperature piezoelectric responses (*d*_33_ > 400 picocoulombs per newton, *k*_33_ > 0.4), surpassing those of existing high-temperature piezoelectrics. Domain-engineered BFHs further unlock programmable control of domain switching, enabling the strongest yet tunable internal bias fields (>8 megavolts per meter) in bulk materials and record-high reversible shear electrostrains (up to 0.98%) at supercoercive field levels. The BFH design concept provides a transformative platform for next-generation ferroelectric and electromechanical transduction devices with extended temperature, high-power, and high-stress capability. It also establishes a universal framework for programmable control across ferroic families, including ferroelastic, ferromagnetic, and multiferroic materials in polycrystalline, single-crystal, and thin-film forms.

## INTRODUCTION

Conventional ferroelectric and piezoelectric materials underpin technologies as diverse as electromechanical sensors, actuators, energy harvesters, and nonvolatile memories. Their deployment in high-temperature, high-voltage, high-power, and high-stress environments, such as aerospace, energy transduction, and advanced manufacturing, still remains critically constrained by intrinsic thermal, electrical, or mechanical depolarization and the instability of engineered domain structures (*1*). In bulk ferroelectrics, collective domain switching enables multifunctionality but is accompanied by undesirable hysteresis, irreversibility, and limited access to a relatively narrow range of ferroelectric domain textures. These intrinsic restrictions suppress strain output, stability, and energy efficiency, preventing bulk ferroelectrics from reaching their theoretical performance limits (*2*, *3*).

Epitaxial heterostructures and superlattices have been exploited for their ability to engineer interfacial strain, valence mismatch, and nanoscale compositional modulations, which can stabilize unconventional polarization states, yielding enhanced transition temperatures and emergent phenomena such as charged domain walls, multiferroicity, and complex polar topologies (*4*, *5*). However, these effects remain confined to ultrathin films, which are fundamentally constrained by substrate compatibility and exhibit limited active volume, poor scalability, and restricted operating conditions (*6*, *7*), in comparison to their scalable and machinable bulk counterparts. A long-standing challenge is therefore to translate the unique interfacial functionalities of heterostructures into robust bulk materials that combine nanoscale design freedom with macroscopic performance. An alternative approach to creating heterostructure-like nanoscale functionality is spinodal decomposition, in which a metastable solid solution separates by continuous uphill diffusion into coherent compositional waves rather than by nucleation and growth of isolated precipitates (*8*). Such self-organized oxide nanostructures have been reported in nonferroelectric systems such as TiO_2_-SnO_2_ ceramics and TiO_2_-VO_2_ rutile oxides (*9*). However, reports in ferroelectric oxides remain very limited, especially for chemically driven spinodal decomposition deliberately controlled by ferroelectric boundary conditions.

In the present study, we report the creation of bulk ferroelectric heterostructures (BFHs) via a scalable, bottom-up route that translates thin-film heterointerface physics into monolithic bulk ferroelectrics via self-organized, spinodal elemental partitioning induced by simple heat treatment. The resulting percolating chemical networks create static ionic bound-charge potentials and coupled ferroelectric-electrostatic-elastic fields that imprint and stabilize domain textures with record yet tunable internal bias. These enable programmable and reversible domain switching and supercoercive electromechancial operation with simultaneously enhanced high-temperature robustness and performance in lead-free ferroelectrics.

## RESULTS

### Creation of BFHs

Herein, we bridge the gap with BFHs, monolithic ferroelectrics in which nanoscale elemental partitioning creates percolating, compositionally modulated networks that act as built-in heterointerfaces (*10*, *11*). The exemplar 0.7BiFeO_3_-0.3BaTiO_3_ (BF-BT) ceramic samples were prepared by conventional solid solution reaction (further details given in the Materials and Methods section), followed by heat treatment to generate BFH. Transmission electron microscopy (TEM) analysis of the ceramic at different stages of the heat treatment process shows profound changes in the microstructure for both unpoled as-sintered (AS) and poled air-quenched (AQ) samples (Fig. 1, B and D) after thermal aging (Fig. 1, C and E). For the case of AS samples, annealing for 200 hours at 500°C (above the Curie temperature of the AS sample, 458°C; Fig. 1A) induced the formation of a relatively disordered domain configuration with underlying nanoscale labyrinthine features (Fig. 1C and fig. S1), identified here as BFHs with random local field (Fig. 1G). The corresponding ferroelectric polarization–electric field (*P*-*E*) hysteresis loop of the AS BF-BT ceramic evolved into a slim, pinched loop for BFH-AS after thermal aging, consistent with internally biased hard-ferroelectric behavior arising from aging-induced nanoscale elemental partitioning and associated local internal fields (Fig. 1F). In contrast, the relatively large domain size and preferred orientation obtained after poling of the AQ (normal FE) sample (Fig. 1D) was retained in the poled BFH-AQ ceramic after thermal aging below *T*_C_ (Fig. 1A), but the uniformity of the chemical composition and the polar ion displacements within each domain were disturbed by locally developed wavelike nanoscale BFH patterns induced by the aging process (Fig. 1E and fig. S2). The corresponding *P*-*E* loop of the poled BFH-AQ ceramic is markedly asymmetric and shifted along the electric field axis, indicating an internal bias field (*E*_ib_ = 7.5 MV m^−1^) of unprecedented strength, in comparison with the saturated but symmetric (*E*_ib_ = 0) *P*-*E* loop of the AQ (normal FE) samples. From this work, three different types of ferroelectric characteristics and distinct polarization morphologies were achieved by controllable bottom-up synthesis of BFH, by modifying the heat treatment parameters of a single BF-BT solid solution and without the need for conventional compositional modification or interfacial engineering.

**Fig. 1.**
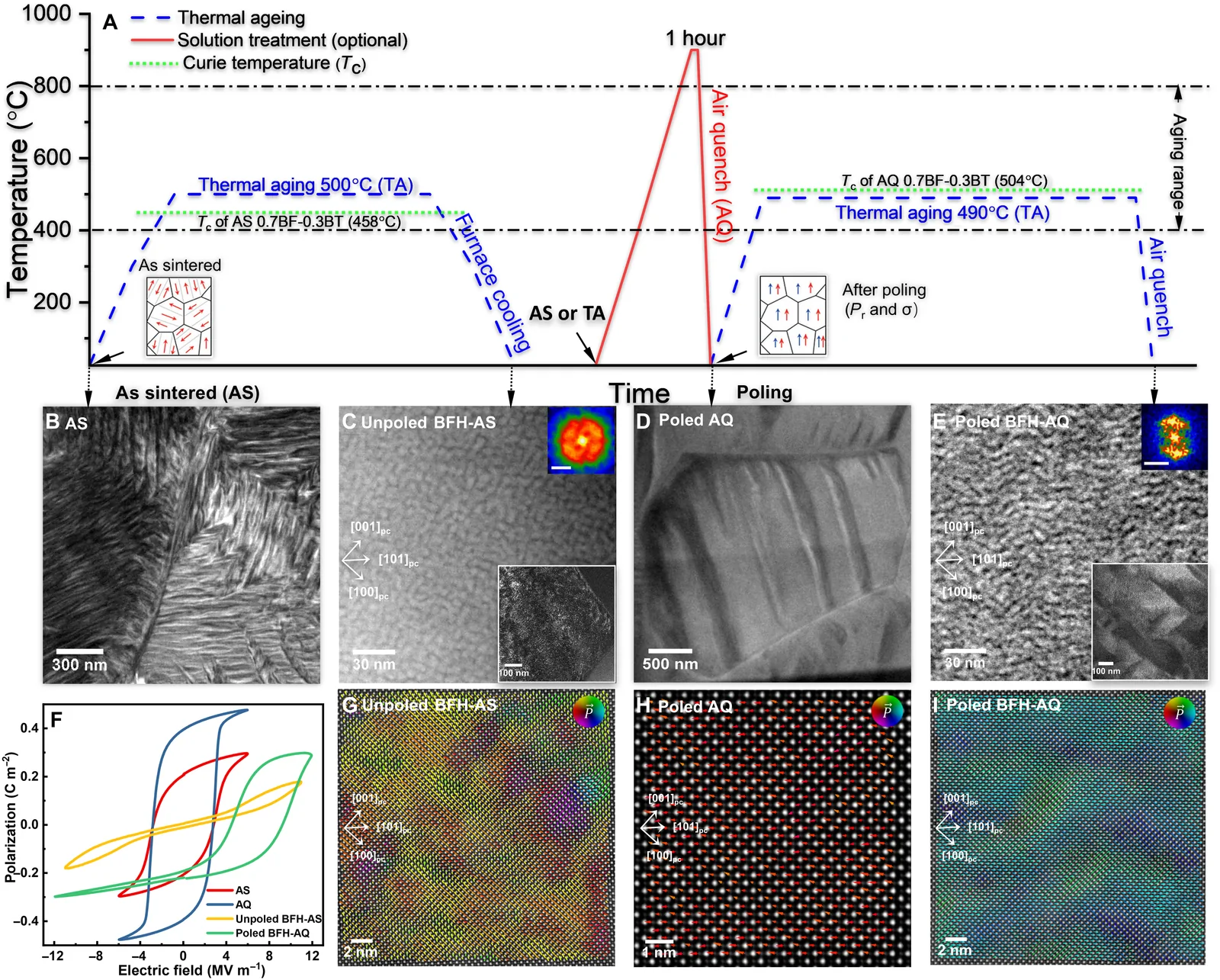
Heat treatment procedures and the corresponding evolution of microstructure and ferroelectric behavior for 0.7BF-0.3BT BFH ceramics. (**A**) Temperature profile of the heat treatment consisting of thermal aging (400° to 800°C) and solution treatment followed by air quenching (1-hour dwell at 900°C followed by rapid cooling to room temperature). Additional poling by an applied electric field can be used before thermal aging to introduce a remanent polarization (*P*_r_) state and associated residual stress (σ). (**B** and **D**) Bright-field TEM images of AS and poled AQ ceramics (normal ferroelectrics) showing lamellar domain structures. (**C**) HAADF image of BFH-AS ceramics viewed along the [010]_pc_ zone axis, revealing the development of modulated nanoscale features with atomic number (*Z*) contrast variation; the top inset shows the FFT of (C), and the bottom inset shows the dark-field TEM image of local polar nanoregions. (**E**) HAADF image of poled BFH-AQ ceramics viewed along the [010]_pc_ zone axis, revealing the development of orientated wavelike patterns within a single domain; the top inset shows the FFT of (E), and the bottom inset shows the retention of broad domain structures after thermal aging in the poled BFH-AQ sample. (**F**) *P*-*E* loops of AS, AQ, BFH-AS, and poled BFH-AQ BF-BT ceramics corresponding to the microstructures shown in (B) to (E). (**G** to **I**) Atomic-resolution HAADF images with the overlaid atomic polarization mappings of BFH-AS, AQ, and poled BFH-AQ samples.

Notably, the high-temperature air quenching process shown in Fig. 1A is used to promote a more homogeneous normal ferroelectric state before thermal aging and to minimize uncontrolled elemental partitioning during slow cooling after sintering. However, this step is not essential for BFH formation. Thermal aging of AS, slow-cooled BF-BT ceramics can also generate BFH microstructures, although the initial microstructural and ferroelectric states differ from those obtained after air quenching. The crack-free microstructure and enhanced fracture toughness ($K_{\text{IC}}$) of AQ samples after quenching (fig. S3) indicates that this processing route is feasible for BF-BT ceramics and can therefore be used as a controlled precursor state for subsequent BFH formation. We note that thermal shock fracture initiation is primarily governed by the competition between thermally induced stress and fracture strength, whereas $K_{\text{IC}}$ is relevant to the propagation and arrest of thermal shock cracks (*12*). The thermal stresses induced by quenching could be further mitigated by reducing the magnitude of the temperature change during quenching (e.g., by rapid cooling selectively over the temperature range from 900° to 400°C) and further released during subsequent thermal aging. The application of the same approach to other solid solutions of BiFeO_3_-SrTiO_3_ and Na_0.5_Bi_0.5_TiO_3_-NaNbO_3_ (fig. S4) also serves to illustrate the potential of this novel approach for materials engineering, both in BiFeO_3_-based systems and in a wide range of other related electroceramic solid solutions that may exhibit tendencies for immiscibility.

High-angle annular dark-field scanning transmission electron microscopy (HAADF-STEM), energy-dispersive x-ray spectroscopy (EDX) and electron energy-loss spectroscopy (EELS) imaging studies were conducted to further investigate the nanoscale patterns observed in TEM images of the BFH-AS and BFH-AQ samples. The combination of imaging and spatially resolved spectroscopic data reveal that these nanoscale patterns relate to local elemental partitioning of regions with Ba^2+^ and Bi^3+^ segregation as shown in the HAADF-STEM *Z*-contrast images (Fig. 1, C and E). The elemental partitioning occurs within a coherent perovskite lattice structure extending across the whole region (figs. S1D and S2D). In comparison with the long-range ferroelectric ordering in the AQ sample with rhombohedral symmetry (Fig. 1H), the presence of aliovalent elemental partitioning of Ba^2+^ and Bi^3+^ significantly disturbs the A-site ion displacements, related local electric field and polarization continuity in both BFH-AS and poled BFH-AQ samples (Fig. 1, G and I). Atomic-scale polarization mapping of the BFH-AS samples reveals the presence of random local field orientations with continuous polarization rotation (Fig. 1G and fig. S5). In contrast, an alternating convergent and divergent polarization distribution centered along the [111]_pc_ (pseudocubic notation) direction was observed in poled BFH-AQ samples (Fig. 1I and fig. S6), which is consistent with the modulated spatial arrangement of nanoscale patterns as shown in Fig. 1E. The relationship between the polarization orientation distribution and elemental partitioning will be discussed later with further detailed elemental analysis.

The formation of nanoscale patterns with modulated spatial arrangements in both BFH-AS and poled BFH-AQ samples (Fig. 1, C and E) is governed by crystallographic elastic anisotropy (particularly between polar and nonpolar directions) and the requirement for minimization of the coherent lattice strain energy (*13*). The presence of wavelike Bi-enriched and Ba-enriched regions with twofold symmetry was observed in both the AS BFH-AS and poled BFH-AQ samples (Fig. 1, C and E) when viewed along the zone axis [010]_pc_. In the BFH-AS samples, the wavelike patterns exhibit oriented growth with twofold symmetry and cuboidal motifs, tending to align along the [100]_pc_ and [001]_pc_ directions. This type of anisotropy is manifested by the diffuse fourfold side lobes surrounding the central diffraction spot in the fast Fourier transform (FFT) of the HAADF image (top inset of Fig. 1C) (*14*). The observation is consistent with the absence of the long-range ordered domains at the thermal aging temperature of 500°C, which is greater than the Curie temperature (*T*_C_ = 458°C) of the AS samples. In contrast, the crystallographic alignment of the elementally partitioned, phase separated regions in the poled BFH-AQ sample is mainly along [111]_pc_ or [$\bar{1}$11]_pc_ directions (corresponding to [101]_pc_ and [$\bar{1}$01]_pc_ within the plane view) in different parent domains (Figs. 1E and 2, A, B, and E). The enhanced aspect ratio and exclusive <111> growth orientation of the nanoscale pattern within an individual parent domain (Fig. 2A) yields twofold side lobes in the FFT (insets of Fig. 2, B and E), which is attributed to the preferred polarization direction and associated elastic strain induced by prepoling. Furthermore, a characteristic diffuse spinodal ring (*15*) was also evident in the selected-area electron diffraction (SAED) pattern (inset of fig. S2A). The wavelength of the periodic compositional fluctuation or the size of the nanofeatures developed in the present study is around 8 nm according to the evaluation of the spinodal ring in SAED and the positions of the side lobes in the HAADF FFT (fig. S7).

**Fig. 2.**
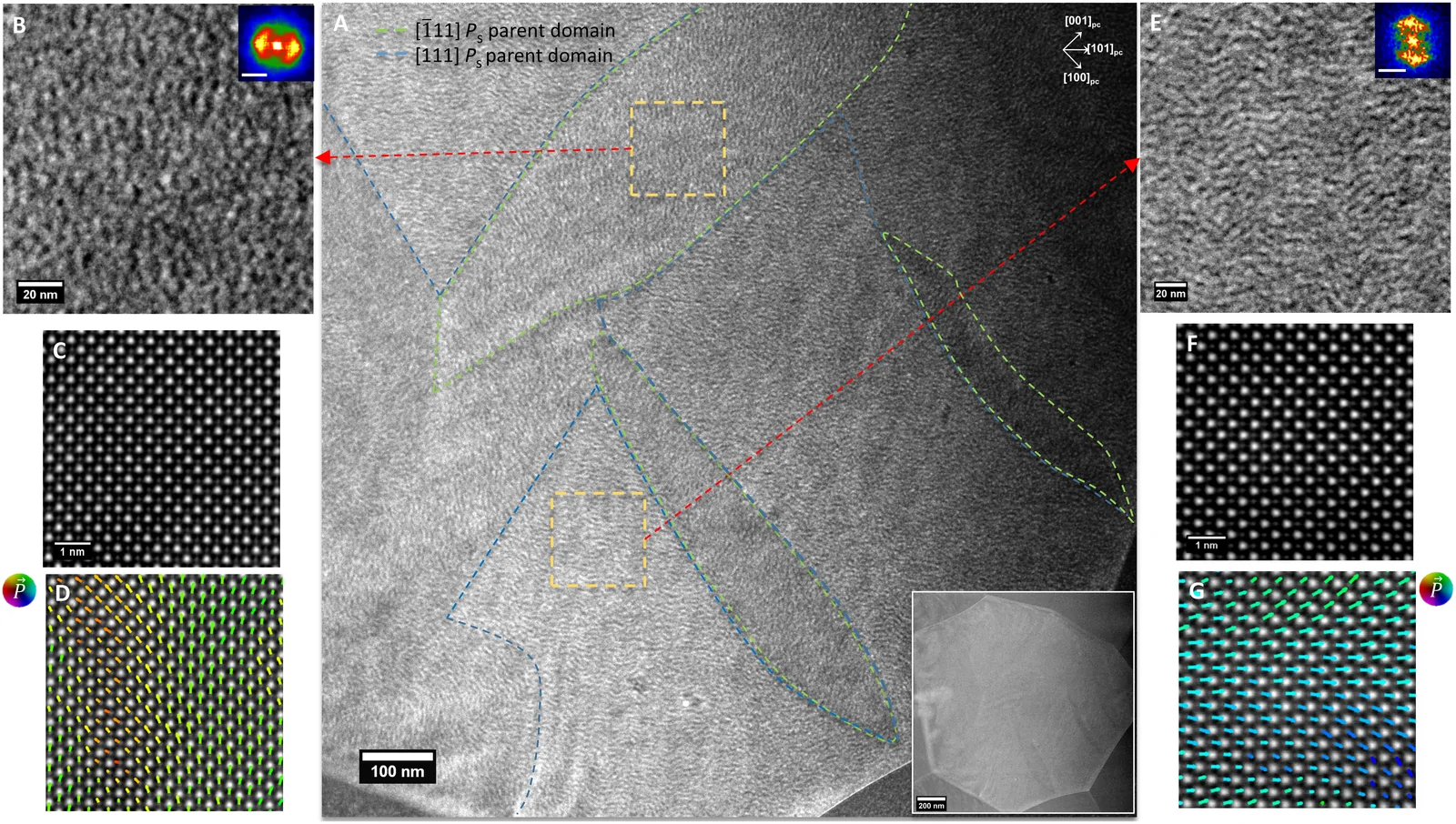
Spatial arrangements of the wavelike features in the 0.7BF-0.3BT poled BFH-AQ ceramic according to the local spontaneous polarization direction. (**A**) HAADF image of the [010] orientated grain contains two types of parent domain variants with polarization along [111]_pc_ and [$\bar{1}$11]_pc_ directions (corresponding to [101]_pc_ and [$\bar{1}$01]_pc_ directions within the plane view, respectively); the inset in (A) shows the full view of the investigated grain. (**B**) HAADF image for the identical region (dashed square) marked in the parent domain with polarization along [$\bar{1}$11]_pc_ in (A), showing the [$\bar{1}$11]_pc_ preferred growth of the wavelike features with associated twofold side lobes along the [111]*_x_* direction in the FFT inset. (**C** and **D**) Atomic-resolution HAADF image and associated polarization vector mapping for the local region in (B) confirm the divergent polarization distribution centered along [$\bar{1}$11]_pc_. (**E**) HAADF image for the identical region (dashed square) marked in the parent domain with polarization along [111]_pc_ in (A), showing the [111]_pc_ preferred growth of the wavelike features with associated twofold side lobes along [$\bar{1}$11]_pc_ in the FFT inset. (**F** and **G**) Atomic-resolution HAADF image and associated polarization vector mapping for the local region in (E) confirm the divergent polarization distribution centered along [111]_pc_. Scale bars, 0.2 nm^−1^ [insets of (B) and (E)].

### Interfacial characteristics of BFHs

Phase-field modeling simulations and HAADF tomographic three-dimensional (3D) reconstruction of the local region in poled BFH-AQ samples reveal the percolation between Ba^2+^-enriched and Bi^3+^-enriched regions by forming cross-linked lamellar networks (Fig. 3, A and E, and movies S1 to S3), which are consistent with the 2D wavelike features viewed along the [010]_pc_ zone axis (Fig. 2A). These results provide the microstructural foundation for the unique macroscopic properties of BFH, demonstrating the characteristics of ferroelectric epitaxial heterostructures or superlattices in a bulk polycrystalline ceramic form. At the interfaces between these elementally segregated structures, the alternating divergent and convergent orientation of projected polarization vectors is modulated together with the alternating enrichment of Ba^2+^ and Bi^3+^, according to the atomic EDX mapping and supported by the phase-field polarization distribution (Fig. 3, B and F). The standardless EDX mapping results (fig. S8) indicate that the amplitude of the A-site concentration variations for Ba^2+^ and Bi^3+^ between the elementally partitioned regions is ∼20 atomic % (at %) (4 at % in total of the ABO_3_ formula unit). In contrast to the pronounced A-site Ba/Bi partitioning, STEM-EDX did not resolve clear segregation of Fe, Ti, or O between the compositionally modulated regions within the sensitivity limits of the measurement (fig. S8). This is partly due to the weaker expected amplitude of B-site variations and, for Ti, the overlap between the Ba Lα_1_ (4.465 keV) and Ti Kα_1_ (4.508 keV) x-ray peaks.

**Fig. 3.**
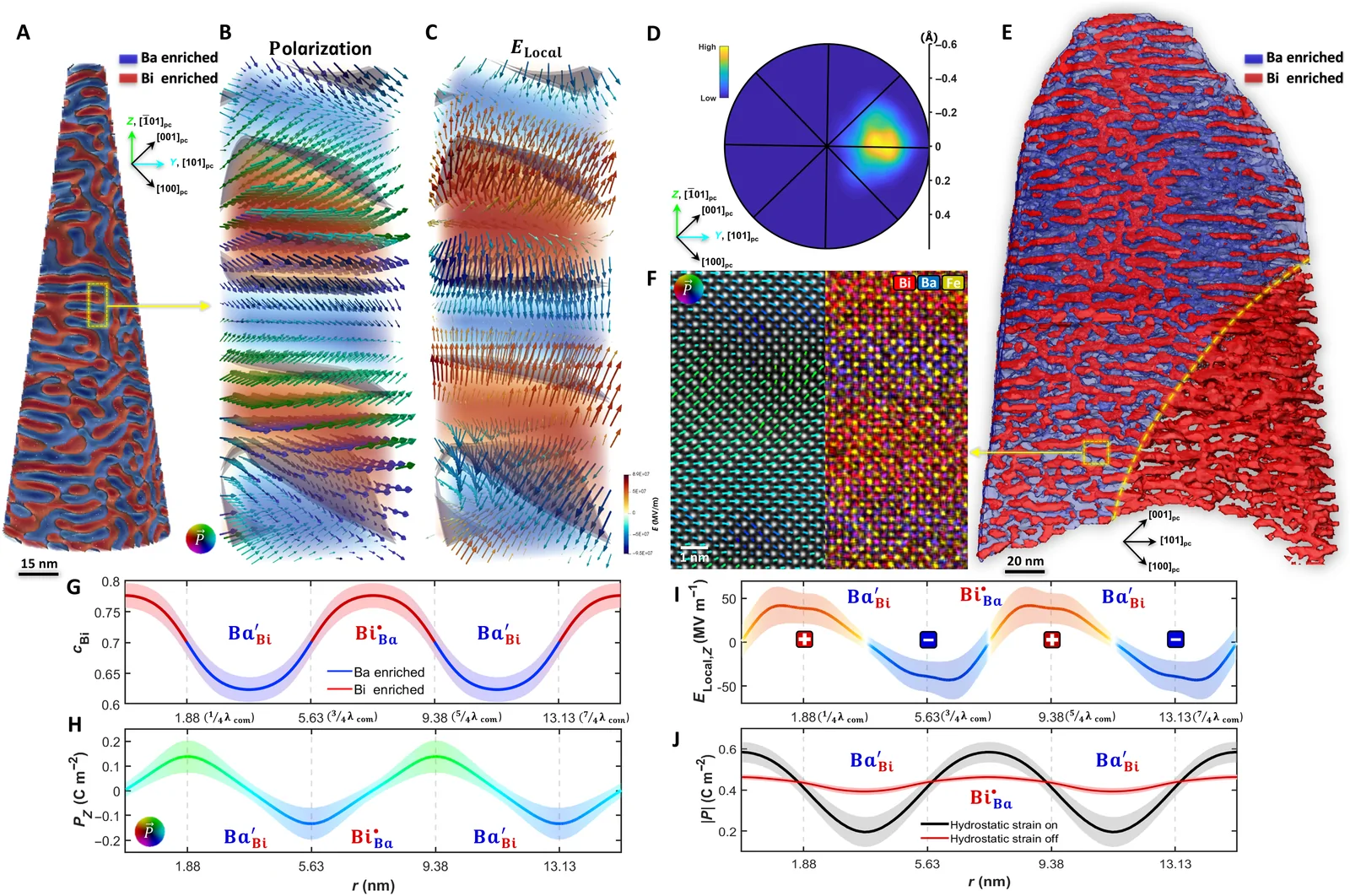
Phase-field modeling and experimental HAADF-STEM characterization of the correlation between local electric field, polarization, and elemental distributions in poled BFH-AQ BF-BT ceramics. (**A**) Phase-field simulated 3D compositional morphology showing cross-linked lamellar networks of Bi-enriched and Ba-enriched regions. (**B** and **C**) Simulated local polarization and electric field vector maps, respectively, taken from the selected region marked by the yellow dashed rectangle in (A). (**D**) Experimental polar plot of the projected polarization vector orientation distribution extracted from the atomic-resolution HAADF-STEM polarization mapping shown in (F). (**E**) Experimental HAADF-STEM tomographic reconstruction showing cross-linked lamellar networks consistent with the simulated morphology in (A). (**F**) Experimental atomic-resolution HAADF-STEM image with EDX mapping and projected polarization vector analysis, showing the spatial correlation between Ba/Bi compositional modulation and local polarization reorientation. (**G** to **J**) Phase-binned profiles extracted from the phase-field simulation, showing the variation of Bi concentration, polarization component along the *z* direction, local electric field component along the *z* direction, and polarization magnitude across two periods of the simulated composition wave $\lambda_{\text{com}}$.

Further evidence for the spatial fluctuations in concentration of Ba, Ti, Fe, and O was obtained by complementary mapping using EELS (Fig. 4). EELS struggled to detect Bi due to the high energy loss and delayed edge structure but provides superior sensitivity for light elements. Analysis of the spectra corresponding to higher and lower intensity regions of the HAADF image confirms the occurrence of Ba elemental partitioning on the nanoscale and also indicates that the Ba enrichment (Bi depletion) is associated with a slight Ti enrichment and Fe depletion (Fig. 4, A to E). The disproportionate B-site elemental segregation only partially compensates the charge imbalance induced by respective aliovalent Ba^2+^ and Bi^3+^ exchange, resulting in the formation of associated ${\text{Bi}}_{\text{Ba}}^{•}$ and ${\text{Ba}}_{\text{Bi}}^{\prime }$ point defects (in Kröger-Vink notation) in adjacent regions. The possible variance of oxidation states for Fe and Ti cations as a charge balancing mechanism is also ruled out by the almost identical chemical shifts and the edge onset EELS spectra for O, Fe, and Ti for both Ba-enriched and Bi-enriched regions (*16*), illustrated in Fig. 4 (F to H). Further complete charge compensation is achieved by the development of alternating divergent and convergent polarization distribution [i.e., charged head-to-head (H-H) and tail-to-tail (T-T) domain wall structures] during thermal aging, which will be further discussed in the following section.

**Fig. 4.**
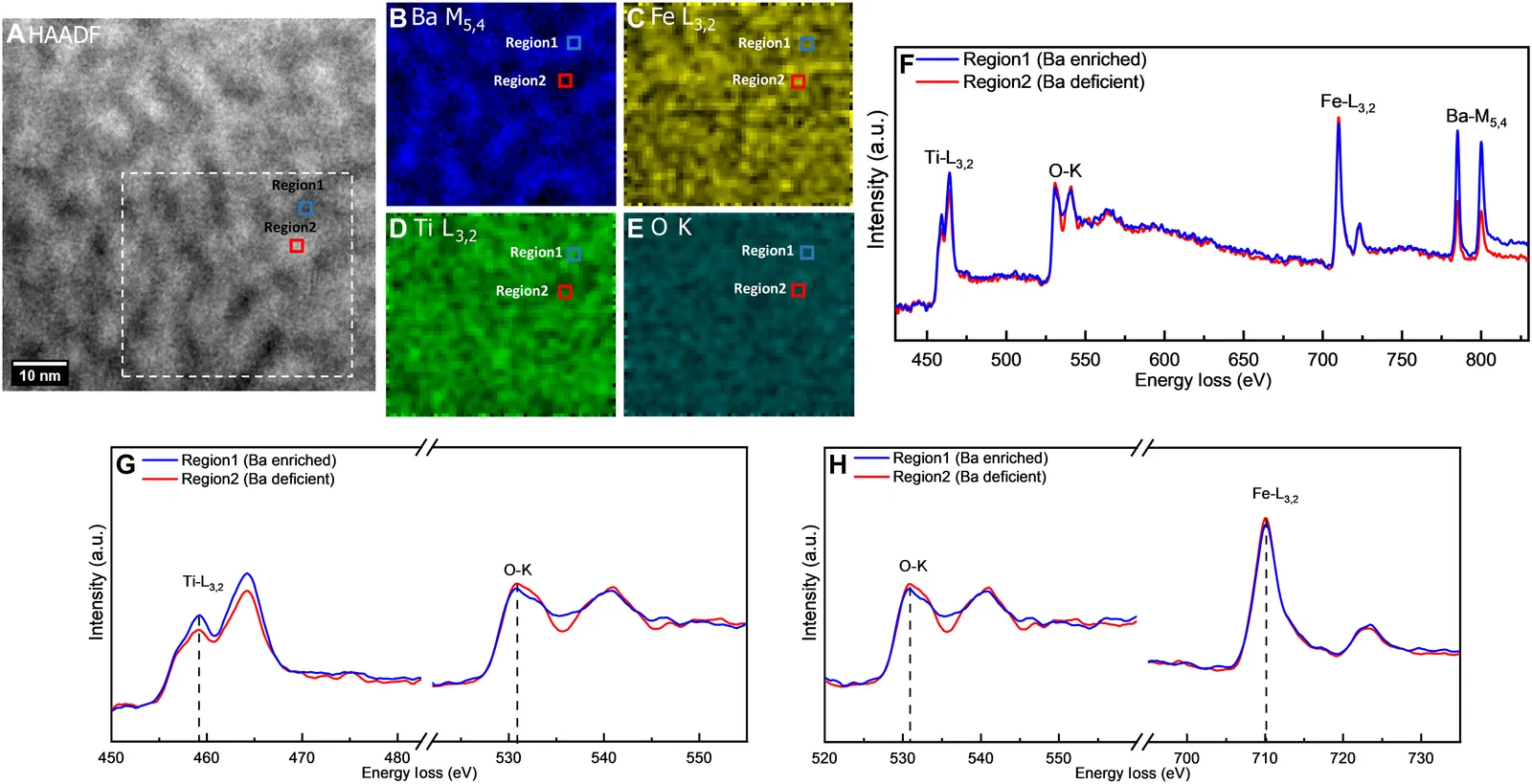
EELS-based elemental mapping of the phase-separated regions in the poled BFH-AQ BF-BT ceramics. (**A** to **E**) HAADF image of the EELS mapped regions (region in dashed rectangle in A) and elemental maps of Ba M_5,4_, Fe L_3,2_, Ti L_3,2_, and O K energy loss edges. (**F** to **H**) Background subtracted EELS from Region1 and Region2 (Ba-enriched and Bi-enriched regions, respectively) labeled in (A). a.u., arbitrary units.

By color indexing the local polarization vectors according to the angle, α, relative to the [101]_pc_ direction, observing the HAADF image intensity variations and mapping the B-site cation displacement magnitude, illustrated in fig. S9 (A to C), it is evident that the local polarization direction periodically deviates from [111]_pc_ in Bi-enriched regions toward [100]_pc_ and [001]_pc_ in neighboring Ba-enriched regions. There is also a pronounced reduction in the magnitude of polarization in Ba-enriched regions (fig. S9B), together with local reorientation, driven by the interfacial and electromechanical coupling between phase-separated regions and hydrostatic strain induced by lattice mismatch, analogous to similar effects in ferroelectric superlattices (*17*). Phase-field simulations validate that the hydrostatic strain drives a pronounced reduction and enhancement in the polarization magnitude, ∣P∣, in Ba-enriched and Bi-enriched regions, respectively (Fig. 3J). By contrast, when hydrostatic strain is not included in the model, ∣P∣ varies only slightly across the Ba-enriched and Bi-enriched regions.

The alternating polarization directions (-[001]_pc_-[111]_pc_-[100]_pc_-[111]_pc_-) are determined by the periodic arrangement of localized antiparallel electric fields (Fig. 3, C and I, and fig. S10B) induced by modulated point defect (${\text{Bi}}_{\text{Ba}}^{•}$ and ${\text{Ba}}_{\text{Bi}}^{\prime }$) enrichment (i.e., valence mismatch) within the [111]_pc_-orientated Ba-enriched and Bi-enriched regions (Fig. 3G), which forms adjacent H-H and T-T domain configurations at the nanoscale along with the associated polarization bound charge (fig. S10C). Additional components of polarization in the vertical (*z*) direction are also evident (Fig. 3, B, F, and H), which counter the ionic charge and local electric fields induced by point defect clustering, thus minimizing the local electrostatic energies and avoiding the so-called polarization catastrophe (*18*).

Overall, the polarization orientation distribution is mainly centered on the [111]_pc_ direction and ranges between [100]_pc_ and [001]_pc_ directions along with the reduction in polarization magnitude in the plane view of Fig. 3 (B and G), which forms a triangular-shaped distribution contour in the polar plot of the polarization vector orientation distribution (Fig. 3D and fig. S6). The observation of charged domain walls in the present study is driven by the elemental segregation of aliovalent Bi^3+^ and Ba^2+^ ions on the A-site sublattice, whereas the H-H and T-T domain configurations reported previously in BiFeO_3_ and PbZr_0.2_Ti_0.8_O_3_ systems are said to be charge compensated by variations in the B-site oxidation state (*19*, *20*). These observations also indicate that the signature random local polarization and electric field distribution (such as that of the unpoled BFH-AS samples shown in Fig. 1G) can be transformed into a modulated local electric field by the combination of elemental partitioning and domain engineering, whereas the charged domain walls are created as a second-order effect of partially compensated aliovalent elemental segregation in addition to phase separation. The static ionic charge potential between immobile Bi^3+^-enriched and Ba^2+^-enriched regions also serves to imprint the local polarization distribution and domain configurations, producing either randomly distributed internal fields with slim, pinched ferroelectric hysteresis loops or macroscopically biased hard-ferroelectric characteristics, depending on whether prepoling is applied (Fig. 1F).

In comparison with the homogeneous state of the AQ sample (Fig. 1H), the crystallographic and compositional analysis results indicate that the formation of the nanoscale patterns with elemental partitioning during thermal aging arise from spinodal decomposition, in preference to the conventional nucleation and growth mechanism. This process is attributed to a miscibility gap within the BF-BT solid solution at the aging temperature, which provides the thermodynamic driving force for spinodal decomposition and selective nanoscale elemental partitioning, primarily through diffusion of A-site cations within the coherent perovskite ABO_3_ lattice. Furthermore, the formation of dual-phase percolation networks (Fig. 3, A and E), lattice coherency (Fig. 3F), and negligible lattice misfit (fig. S11) across adjacent phase-separated regions indicates the development of Eshelby-type constraints (*21*) and opposing hydrostatic stresses between Bi-enriched and Ba-enriched regions, considering the significant discrepancy between the ionic radii of Ba^2+^ (1.61 Å) and Bi^3+^ (1.45 Å, extrapolated value) in 12-fold coordination (*22*). Overall, aliovalent element partitioning in bulk ferroelectrics acts as a compositionally encoded source of internal fields by activating tightly coupled ferroelectric-electrostatic-elastic responses at the nanoscale. The resulting internal fields stabilize and steer polarization and imprint domain configurations, which can be biased or random depending on the electromechanical boundary conditions used to form the BFH. Exploiting the programmable BFH architecture and internal field enables emergent bulk responses not accessible to epitaxial thin films or traditional bulk ferroelectrics, as detailed in the next sections.

### Thermal stability and ferroelectric hardening in BFHs

Despite decades of research, developing piezoelectric materials with high sensitivity and efficiency at elevated temperatures remains a major challenge (*23*, *24*). The need is urgent, driven by applications in online process monitoring, structural health assessment, and electromechanical actuation across energy, petrochemical, mining, metallurgy, automotive, aviation, and aerospace sectors (*1*). For instance, in the EU alone, a 10% reduction in forced power plant outages, achievable with ultrasonic sensors operating for continuous monitoring of superheated steam line welds at 580°C, was estimated to yield annual savings of €2.5 billion (*25*, *26*).

The BF-BT BFH ceramics developed in this study (described in the Materials and Methods section) address this challenge by combining enhanced Curie temperature (*T*_C_ = 824°C; Fig. 5, A and B), exceptional room temperature piezoelectric responses (charge coefficient *d*_33_ = 115 pC N^−1^ and planar coupling factor *k*_p_ = 0.37; Fig. 5A and fig. S12A), and superior sensitivity, outperforming other high-temperature piezoelectrics suitable for operation at 580°C (*27*–*31*). The high-temperature performance of BF-BT BFH ceramics (*d*_33_ > 400 pC N^−1^, *k*_33_ > 0.4, and mechanical quality factor *Q*_m_ > 800; Fig. 5, C and E) is comparable to the room temperature properties of commercial PbZr_(1−*x*)_Ti*_x_*O_3_ (PZT) ceramics. Short-term stability is maintained up to 800°C (Fig. 5D), whereas long-term operation at 580°C shows <3% degradation in *d*_33_ after 1000 hours (fig. S12B). The remarkable increment in Curie temperature for the BFH ceramics is attributed to the high Curie temperature of the Bi-enriched phase within the dual-elemental segregated superstructure, combined with tensile hydrostatic pressure exerted by the surrounding Ba-enriched regions with correspondingly larger unit cell volume (Fig. 5F). This mechanism parallels effects in magnetic spinodal alloys (*32*), is distinct from the biaxial strain–engineered epitaxial heterostructures (*33*), and contrasts with the suppression of *T*_C_ induced by compressive hydrostatic pressure or chemical pressure in BiFeO_3_-based systems (*34*).

**Fig. 5.**
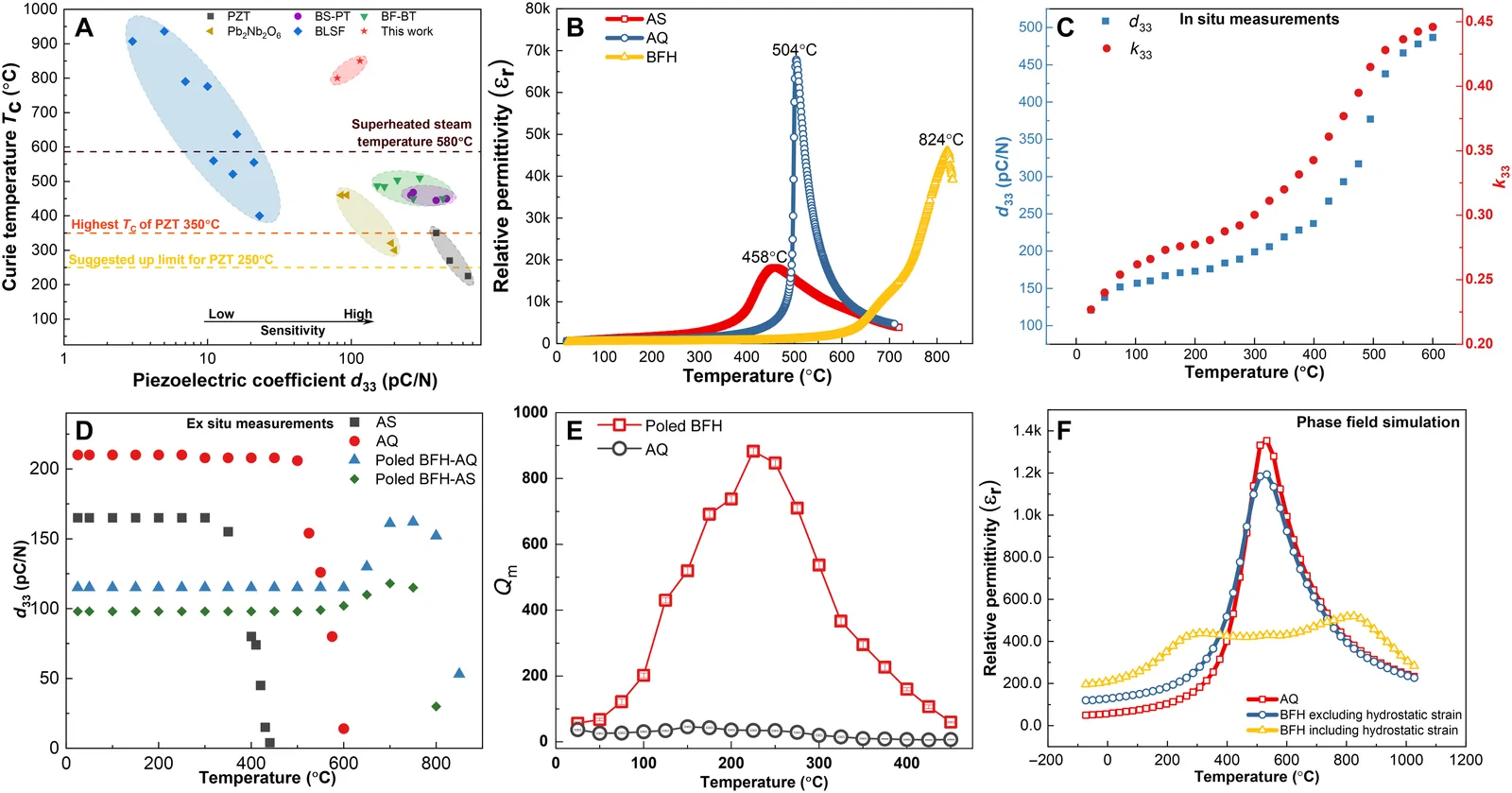
Thermal stability and ferroelectric hardening in BFH. Comparison of the (**A**) piezoelectric coefficient (*d*_33_) between the BFH ceramics and other perovskite and nonperovskite ceramics, including PZT, BiScO_3_-PbTiO_3_ (BS-PT), BF-BT, Pb_2_Nb_2_O_6_, and bismuth layer–structured ferroelectrics (BLSF). (**B**) Temperature dependence of the relative permittivity for AS, AQ, and poled BFH samples. (**C**) Temperature dependence of *d*_33_ and coupling factor (*k*_33_) of poled BFH determined by the resonance method. (**D**) Ex situ measurement of *d*_33_ after dwelling at various temperatures for AS, normal ferroelectric (AQ), and poled BFH samples. (**E**) Temperature dependence of the mechanical quality factor of normal ferroelectric (AQ) and poled BFH samples. (**F**) Temperature-dependent relative permittivity along [111] achieved by phase-field modeling for normal ferroelectrics (AQ) and BFHs exclude and include the contribution of hydrostatic strain using the BF-BT Landau coefficient, respectively.

We further demonstrate the strongest ferroelectric hardening effect reported to date, characterized by an internal bias field exceeding 8 MV m^−1^ (Fig. 6A), nearly an order of magnitude higher than values previously reported in bulk ferroelectrics (*35*). Together with enhanced thermal resilience, this hardening effect directly addresses long-standing limits to high-power, high-temperature operation of piezoelectric transformers, where low coercive fields and self-heating drive thermal and electrical depolarization of conventional ferroelectrics (*36*, *37*). The internal bias field is tunable according to the thermal aging temperature and dwell time (Fig. 6A), providing a versatile route to develop programmable BFH through domain engineering, as demonstrated in the next section.

**Fig. 6.**
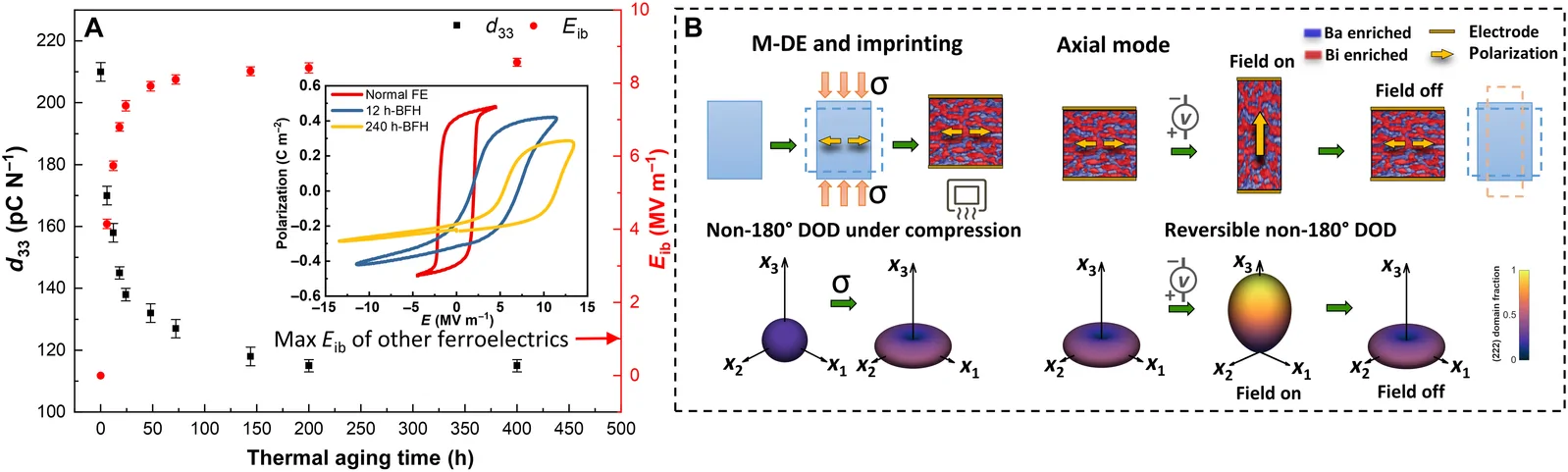
Tunable ferroelectric hardening and mechanical domain engineering and imprinting process. (**A**) Variations of the internal bias field (*E*_ib_), measured at 75°C, and room temperature piezoelectric coefficient (*d*_33_) in prepoled BFH ceramics as a function of the duration of thermal aging at 470°C. h, hours. (**B**) M-DE BFH ceramics with imprinted DOD, exploiting the internal bias field via thermal aging as shown in (A). The non-180° DOD surfaces presented in (B) were determined by in situ SXRD experiments.

### Programmable domain engineering in BFHs

Ferroelectric texture, quantified in terms of the domain orientation distribution (DOD), can be rapidly rewritten when an applied electric field approaches or exceeds the coercive field, producing characteristic hysteresis (fig. S13A). Consequently, ferroelectric texture is inherently transient compared with the persistent crystallographic texture established by conventional texture engineering (*38*). Operation under subcoercive field levels preserves piezoelectric functionality, whereas higher fields induce depolarization, cross-poling, actuator drift, and hysteresis (fig. S13, B and C), thereby limiting strain level, energy density, and actuation precision (*39*, *40*). In principle, reversible domain switching could unlock supercoercive field operation for enhanced domain switching–driven functionalities while allowing the recovery of preset ferroelectric textures. However, the existing approaches to achieve reversible domain switching, such as oxygen vacancy imprinting or heterointerface design, either suffer from instability under electrical and thermal input (*41*, *42*) or are restricted to nanoscale films, presenting a major challenge for achieving the complete control of domain switching behavior in bulk ferroelectrics.

Here, we address this gap by exploiting internal fields induced by cation element partitioning to create programmable ferroelectrics in which the domain configuration (ferroelectric texture) can be deliberately imprinted, tuned, and reset in a controllable and reversible manner, such that the functional response (e.g., strain, polarization, electrical conductivity, and thermal transport) can be predefined, reconfigured, and recovered on demand. In the exemplar mechanical and electrical domain-engineered BFHs (M-DE BFH and E-DE BFH, respectively), axial (poled), and in-plane (mechanically depoled) ferroelectric textures are predefined by electrical field or mechanical stress, respectively, and then “locked in” by nanoscale elemental partitioning (sample preparation details given in the Materials and Methods section). These ferroelectric textures can be recovered after the removal of the external supercoercive electrical or mechanical loads, yielding enhanced axial and shear electromechanical responses.

In the axial mode, M-DE BFH ceramics (Fig. 6B) can experience nearly the full ferroelectric texture range by reversibly switching between in-plane and axial ferroelectric textures under external field cycling, revealed by the in situ x-ray diffraction (XRD) data presented in Fig. 7 (A to C). These results yield a domain switching fraction (Δη_222_) up to 0.88 and reversible fractions as high as 0.77, which is an order of magnitude higher than that of normal FE (Fig. 7J). This leads to “digital-like” electrostrain responses, stable constricted *P*-*E* loops, and large recoverable electrostrains (*S*_3_ up to 5.2 × 10^−3^ at 5 MV m^−1^; Fig. 7, I and K). The field of 5 MV m^−1^ should therefore be regarded as a laboratory high-field condition for demonstrating reversible supercoercive actuation, not as a universal safe operating field for devices. Practical implementation will require optimization of geometry, electrode design, breakdown strength, heat dissipation, and fatigue lifetime, whereas the central advantage of BFH remains the combined enhancement of reversible domain engineering, internal bias, Curie temperature, and high-temperature piezoelectric response.

**Fig. 7.**
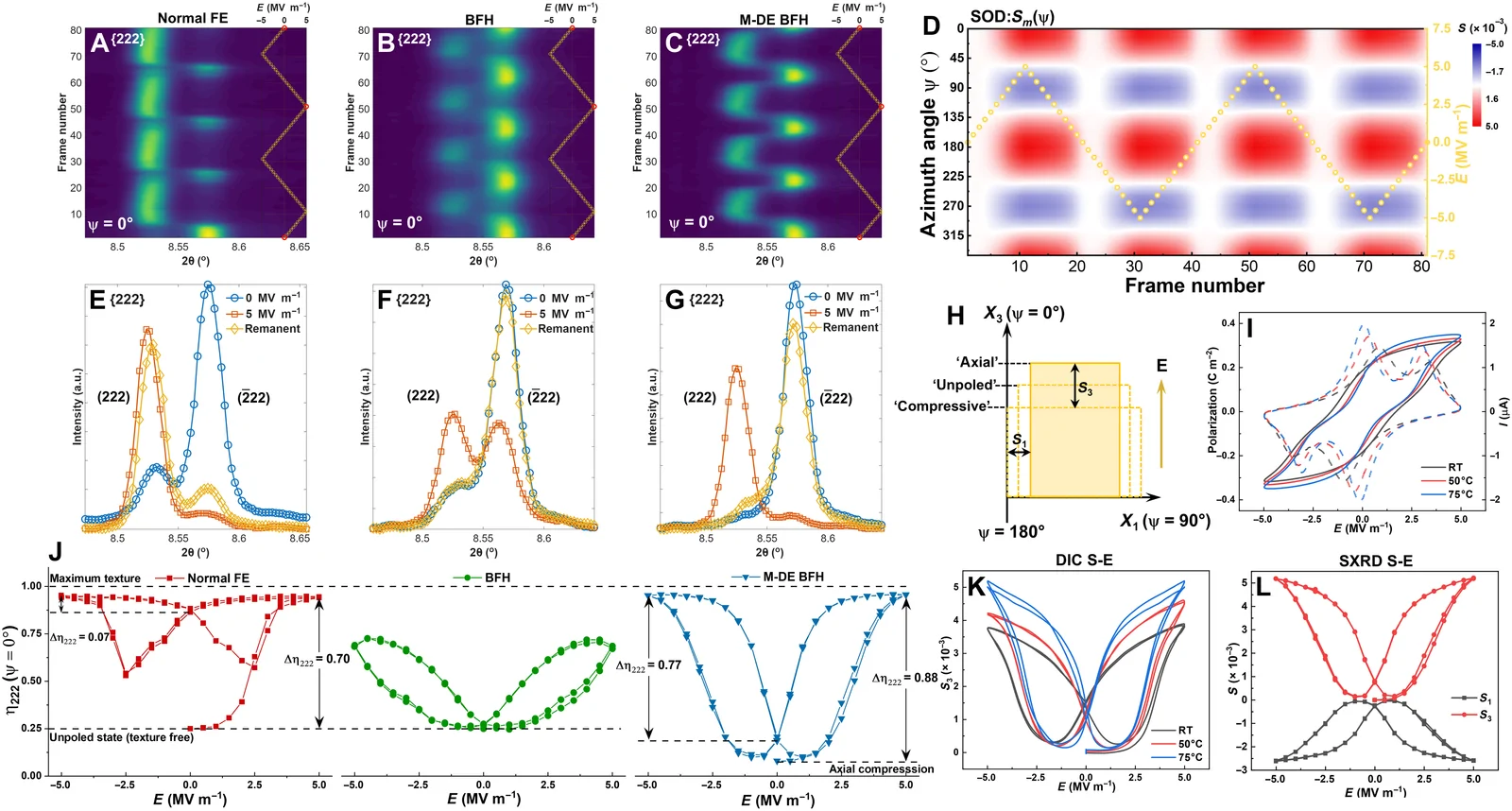
Axial electromechanical behavior of M-DE BFH ceramics, determined by in situ SXRD. (**A** to **C**) Contour plots of XRD peaks for the evolution of {222} reflections in the axial direction (ψ = 0°) under the bipolar electric field for normal ferroelectric (FE), BFH, and M-DE BFH ceramics at 75°C. (**D**) Evolution of the azimuthal angle–dependent macroscopic strain distribution (SOD), *S_m_*(ψ), of M-DE BFH ceramics with the applied electric field at 75°C. (**E** to **G**) In situ XRD profiles of {222} reflections in the initial state without field (0 MV m^−1^), for an electric field of 5 MV m^−1^, and in the remanent state for normal FE, BFH, and M-DE BFH ceramics, respectively, selected from the contour plots in (A) to (C) and highlighted by red circles in the profile of electric field waveforms. (**H**) Illustration of the electrostrain behavior of M-DE BFH and the definition of global axes. (**I**) Polarization and current–electric field (*P*/*I*-*E*) loops of M-DE BFH ceramics. RT, room temperature. (**J**) Electric field–dependent domain fraction of (222) poles, η_222_, for normal FE, BHF, and M-DE BFH ceramics. (**K**) *S*-*E* loops of M-DE BFH ceramics measured by the DIC method. (**L**) *S*-*E* loops of M-DE BFH ceramics evaluated by in situ SXRD results acquired at 75°C.

Quantitatively, the enhanced electrostrain arises from the recovery of the imprinted (mechanically depoled) compressive strain of −1.75 × 10^−3^ (fig. S14A) from the field-induced maximum peak to peak axial strain of 3.5 × 10^−3^ in the normal FE 0.7BF-0.3BT (fig. S14B) under a near-saturated DOD state. The reversibility of domain switching and the associated electromechanical behavior were also confirmed by macroscopic digital image correlation (DIC) strain measurements, showing good correlation with the total strain deduced from analysis of the in situ XRD data (Fig. 7, K and L). This design concept could be further extended to programmable control of thermal transport, electrical conductivity, and electrocaloric responses in ferroelectrics (*43*–*45*), thereby expanding the accessible performance space and enabling “digital-like” switching behaviors.

For operation in the shear mode, an internal bias field of 1.5 MV m^−1^ was created along the transverse direction (*X*_1_) of an E-DE BFH ceramic (Fig. 8A) by annealing at 470°C for 3 hours (global axes defined in Fig. 8A). This resulted in a shifted asymmetric transverse *P*_1_-*E* loop and a constricted symmetric axial *P*_3_-*E* loop (Fig. 8B), indicating the occurrence of reversible domain switching from the *X*_3_ to *X*_1_ direction upon field removal. The resulting shear strains (*S*_5_), measured by DIC, reached 4.25 × 10^−3^ (unipolar) and 8.5 × 10^−3^ (bipolar, peak-to-peak) as shown in Fig. 8C and movies S4 and S5, exceeding the maximum shear strains (*S*_5_) achieved in conventional soft or hard PZT by more than fourfold (*46*). Such shear mode operation is generally inaccessible in thin-film devices, which are limited to purely in-plane or out-of-plane axial actuation modes. The attainable shear strain in E-DE BFH ceramics is also maximized by the recovery of domain switching from the “field-on” state (azimuthal angle, ψ = 45°) back to the *X*_1_ direction upon removal of the applied electric field, overcoming the limitation of memory effects from re-poling or cross-poling and yielding pinched *S*_5_-*E* loops (Fig. 8C) with “digital-like” displacement behavior.

**Fig. 8.**
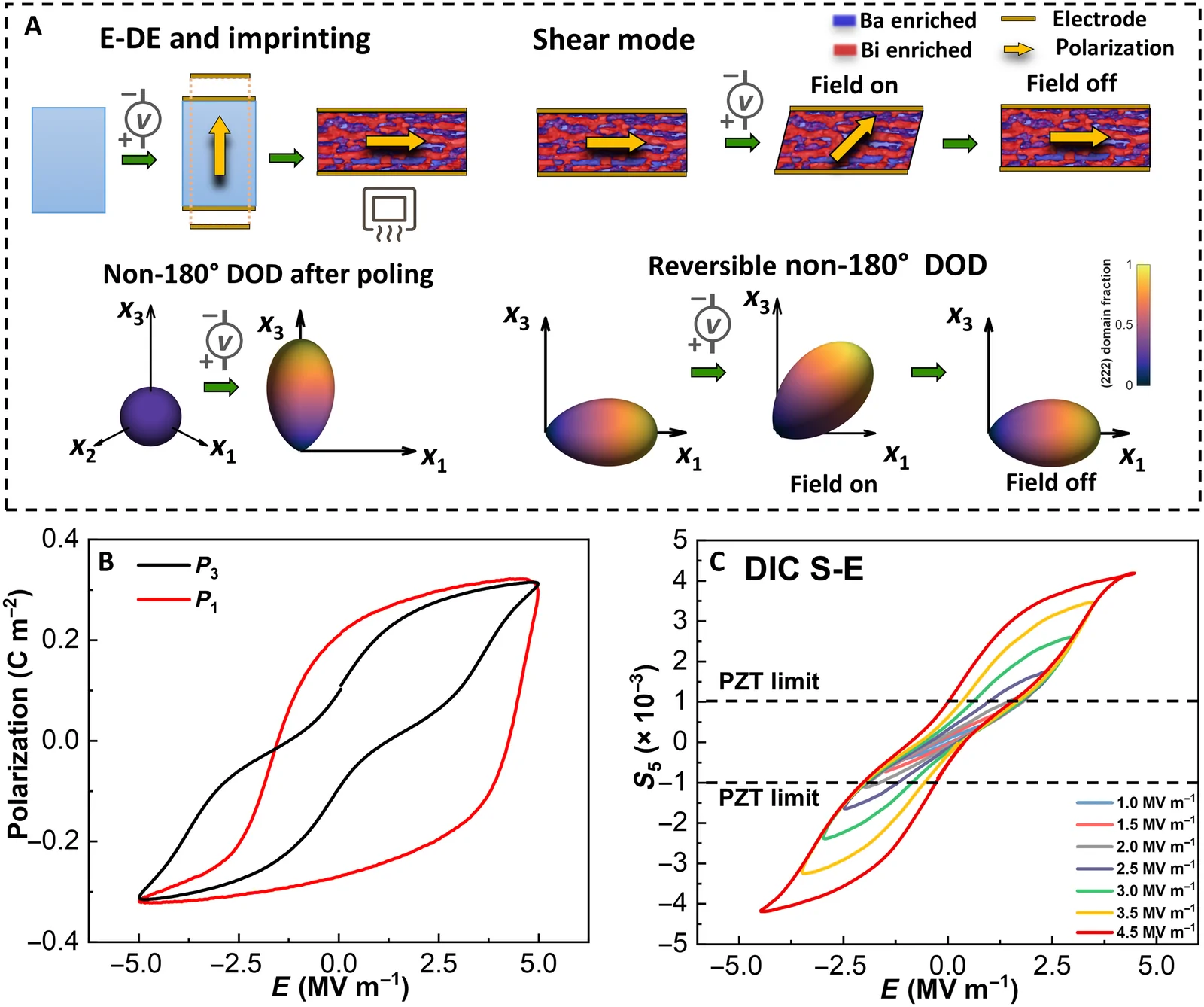
Electrical domain engineering for the application of reversible shear actuation mode. (**A**) E-DE BFH ceramics with imprinted DOD, exploiting the internal bias field obtained via thermal aging (Fig. 6A). The non-180° DOD surfaces presented in (A) were determined by in situ SXRD experiments. (**B**) *P*-*E* loops of E-DE BFH ceramics measured from axial (*P*_3_*-E*) and transverse (*P*_1_-*E*) directions. (**C**) *S*_5_-*E* loops measured under different field levels up to 4.5 MV m^−1^ at room temperature.

For BiFeO_3_-BaTiO_3_ solid solutions, it was observed that the aliovalent elemental partitioning process is most prominent at elevated temperatures between 440° and 800°C. Below this temperature range, the A-site cations (Bi^3+^ and Ba^2+^ in this case) are essentially immobile. Consequently, the BFH 0.7BF–0.3BT ceramics do not suffer from the electric field–driven “deaging” effects that are commonly observed in acceptor-doped hard ferroelectrics, in which the stabilization of domain walls is achieved by the reorientation of relatively mobile acceptor ion-oxygen vacancy defect associates (*41*, *42*). Therefore, the electric field–induced shear strain in BFH 0.7BF–0.3BT ceramics exhibits excellent stability under either unipolar or bipolar field cycling and shows no evidence of deaging effects at either room or elevated temperatures, as shown in Fig. 9B and fig. S15 (A to C). The shear mode electromechanical performance is further enhanced with increasing temperature; the highest effective converse shear piezoelectric coefficient, $d_{15}^{*}$, increases from about 1000 to 1370 pm V^−1^, whereas the peak to peak shear strain rises from 8.5 × 10^−3^ to 9.8 × 10^−3^ at room temperature and 100°C, respectively (Fig. 9C and fig. S15C). The reversible domain switching also ensures the retention of the low-field shear piezoelectricity after exposure to an electric field above the coercive field, *E*_c_ (fig. S15D), in comparison with the loss of shear strain in normal ferroelectrics after cross-poling (fig. S13B). The reversibility of shear strain can be further tuned by modifying the level of the internal bias field by the selection of a different thermal aging time (fig. S15, E and F).

**Fig. 9.**
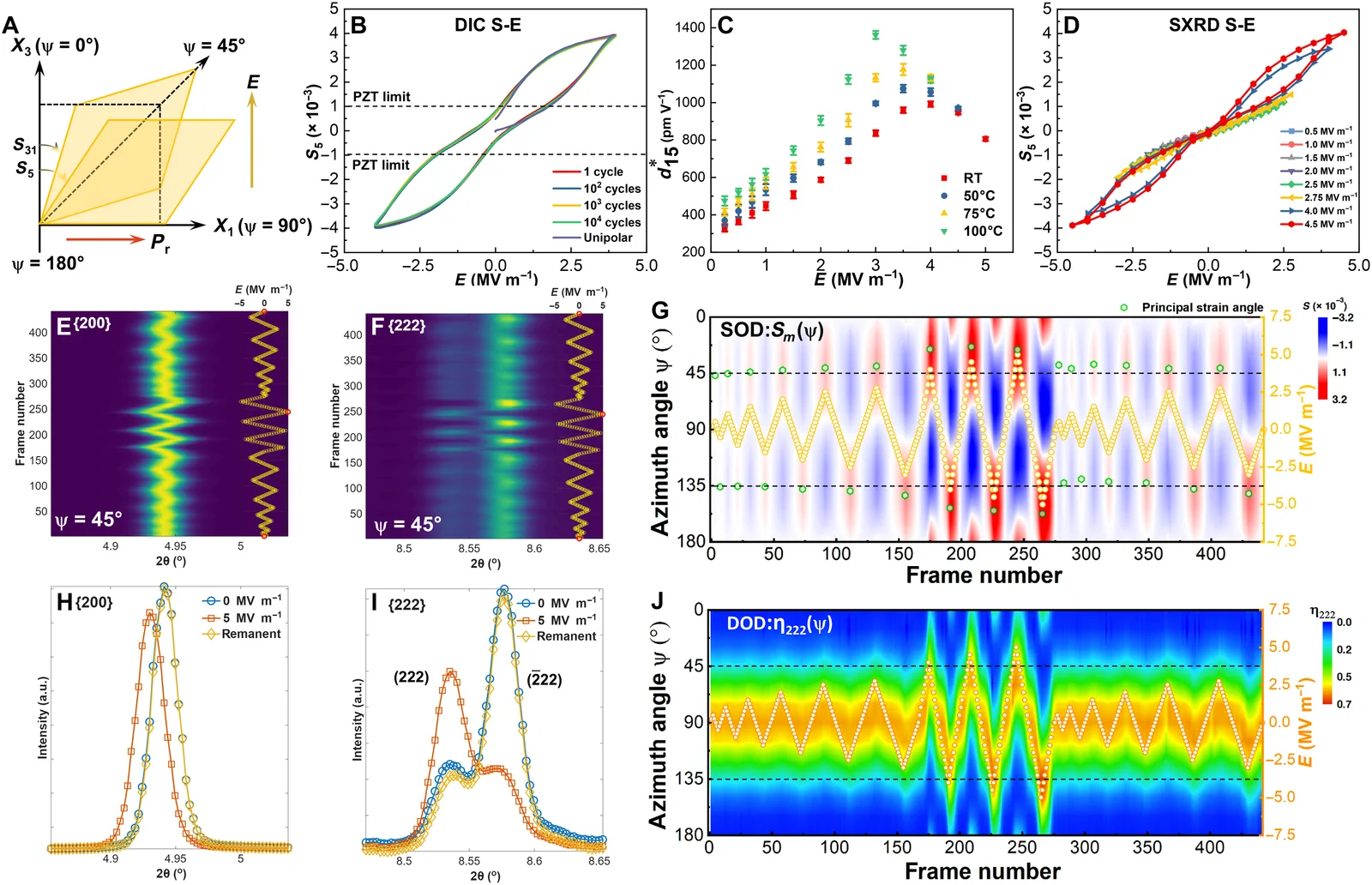
Shear electromechanical behavior of E-DE BFH ceramics. (**A**) Illustration of the relationship between engineering shear strain (*S*_5_) and tensorial shear strain (*S*_31_) with respect to the definition of global axis and remanent polarization direction. (**B**) Repetition of the *S*_5_-*E* measurements at 4 MV m^−1^ after the samples were subjected to a bipolar electric field of 5 MV m^−1^ for one cycle and 10^2^, 10^3^, and 10^4^ cycles, with an additional unipolar *S*_5_-*E* measurement after the test. (**C**) Electric field–dependent effective shear piezoelectric coefficient, $d_{15}^{*}$, of E-DE BFH ceramics measured from room temperature to 100°C. (**D**) *S*_5_-*E* loops measured by in situ SXRD at field levels up to 4.5 MV m^−1^. (**E** and **F**) Contour plots for the evolution of {200} and {222} reflections for E-DE BFH ceramics for azimuthal angle, ψ = 45° (shear direction), under a bipolar electric field waveform with progressively increasing magnitude. Comparison of selected XRD profiles for (**H**) {200} and (**I**) {222} in the initial zero-field state (0 MV m^−1^), at an electric field of 5 MV m^−1^, and in the remanent state. (**G** and **J**) Evolution of the azimuthal angle–dependent SOD and DOD for the respective macroscopic strain, *S_m_*(ψ), and the domain fraction, η_222_(ψ), under a bipolar electric field waveform with progressively increasing amplitude.

Further in situ synchrotron x-ray diffraction (SXRD) measurements were performed to validate the DIC strain data and elucidate the micromechanical origin of the electric field–induced shear strain in E-DE BFH 0.7BF–0.3BT ceramics. In this work, an azimuthal angle of ψ = 45° was selected to indicate the development of lattice shear strain by the shift of the singular {200} peak, whereas variations in the relative intensities of the (222) and ($\bar{2}$22) reflections, corresponding to the polar and nonpolar directions, respectively, provide the means to quantify the degree of non-180° domain switching (Fig. 9F). Pronounced domain switching behavior was observed only for electric field levels above 2.5 MV m^−1^. Below *E*_c_, the electromechanical strain is dominated by the intrinsic piezoelectric effect, giving rise to lattice strain with negligible domain switching (Fig. 9, G and J). The complete reversibility of non-180° domain switching is confirmed by the almost identical peak profiles of {200} and {222} reflections before and after the exposure to an electric field of 5 MV m^−1^, which is significantly greater than *E*_c_ (Fig. 9, H and I). Although the orientation of principal strain, ψ_p_, deviates from the shear direction (ψ = 45°) above *E*_c_ (Fig. 9G), it still contributes significantly to the improved shear strain tensor component, *S*_31_, via enhanced domain switching along ψ = 45° as shown in the DOD for the polar (222) direction in Fig. 9J (details for quantification of DOD and principal strain given in the Materials and Methods section). The estimated field-induced shear strain *S*_5_ (*S*_5_ = 2*S*_31_; Fig. 9, A and D), extracted from the strain orientation distribution (SOD) (ψ = 45°), agrees well with DIC results across the range of electric field levels (Fig. 8C), confirming the reliability of both DIC and SXRD measurements.

## DISCUSSION

In summary, the creation of BFH bridges the gap between ferroelectric epitaxial heterostructures and bulk ferroelectrics. The exploitation of interfacial valence and lattice mismatch turns immiscibility and element partitioning into a programmable engine for emergent functionality in bulk ferroelectrics. By the application of straightforward heat treatment procedures, without modification of composition, BFHs (i) impose internal fields that induce either long-range ferroelectric order, relaxor-like short-range order, or biased hard-ferroelectric states, (ii) control polarization continuity via locked-in ionic bound charge, (iii) stabilize charged domain walls as a second-order effect of aliovalent cation partitioning, (iv) exploit opposing hydrostatic stresses to elevate Curie temperature and enhance piezoelectric performance for high-temperature applications, (v) exhibit tunable ferroelectric hardening effects of unprecedented strength for high-power energy transduction, (vi) expand the functional operating space through programmable switching between different ferroelectric texture configurations, and (vii) enable supercoercive field operation that boosts performance without erasing the preset ferroelectric texture. Notably, the complementary phase-field simulations performed in this work provide a unifying theoretical framework that links spinodal elemental partitioning to the emergence of local chemical heterogeneity, nanoscale polar regions, and their strongly coupled ferroelectric-electrostatic-elastic fields, offering predictive guidelines for designing BFH architectures in a broad class of ferroic materials. By uniting domain engineering with nanoscale self-organization in a scalable, sustainable bulk platform, this approach opens a new operating space for stable, high-temperature, high-voltage, and high-power electromechanical sensors and actuators, transducers and transformers, thermal switches, and electro-optics and offers a general blueprint for unlocking emergent properties across bulk multiferroics, ionic conductors, thermoelectrics, pyroelectrics, and domain wall devices.

## MATERIALS AND METHODS

### Sample preparation by solid-state reaction and high-temperature sintering

(1−*x*)BiFeO_3_-*x*BaTiO_3_ ceramics with various compositions (*x* = 0.2, 0.25, 0.3, 0.35, 0.4) were prepared by solid-state reaction, using commercially available powders of Bi_2_O_3_ (99.9%), Fe_2_O_3_ (99.9%), BaCO_3_ (99.9%), and TiO_2_ (99.9%) (Sigma-Aldrich). The stoichiometric mixture of the precursor powders was planetary ball milled (FRITSCH PULVERISETTE 5) with yttria-stabilized zirconia beads as milling media in propan-2-ol for 6 hours using a rotational speed of 400 rpm. Afterward, the dried powders were calcined at 800°C for 4 hours in air and wet planetary ball milled for a further 6 hours. The calcined powders were dried and uniaxially pressed in a cylindrical steel die at a pressure of 50 MPa. The green pellets were then sintered at temperatures from 1010° to 1100°C (depending on composition) for 2 hours. Planetary ball milling was previously demonstrated as an effective method to suppress the core-shell structure formation along with enhanced homogenization during calcination and sintering. The AQ BF-BT ceramics were prepared by reheating the AS samples to 900°C (above the region of immiscibility), dwelling for 30 min, and then quenching to room temperature in air. The 8 mm–by–1 mm ceramic discs were transferred from a 900°C environment in furnace and placed on a alumina tile at room temperature during quenching, which allows a fast cooling for the samples to reach room temperature within 30 s, given a cooling rate about 30°C s^−1^. Thermal aging was conducted on both AS and poled AQ samples at different temperatures (500° to 800°C) and with various dwell times (12 to 240 hours) to create BFH-AS and poled BHF-AQ samples, respectively. It was considered that an external short circuit was not necessary during thermal aging of the poled sample due to the thermally activated conductivity, providing a pathway to eliminate any pyroelectric potential (*47*). An additional stepwise thermal aging experiment was carried out for poled BFH (AS) samples using a poled AS sample by annealing at 430°C and subsequently at 470°C, with a dwell time of 150 hours for each step. Conductive electrodes were applied using a silver paste (GWENT C2130823D1), fired on at a relatively low temperature of 300°C for 10 min to minimize the thermal influence on the samples.

### M-DE BFH sample preparation

The uniaxial compression and thermal aging experiments on normal FE BF-BT ceramics were performed with the Instron 6800 Series Universal Testing System. The samples were ground and polished to achieve parallel surfaces (accuracy: <3 μm) for thermomechanical experiments. A square-ended beam-shaped sample (10 mm by 5 mm by 5 mm in dimensions) was carefully centered and aligned with the stress axis with the assistance of a centering tool at room temperature. The sample was then heated to 350°C with a heating rate of 10°C min^−1^ to apply a constant compressive stress of 50 MPa. After dwelling for 10 min at 350°C under 50-MPa compressive stress, the temperature was increased to 475°C with a heating rate of 10°C min^−1^, retaining the constant compressive stress of 50 MPa. The sample was then thermally aged at 475°C under 50 MPa compressive stress for 3 hours, followed by a cooling process with a cooling rate of 10°C min^−1^. The M-DE BFH sample achieved via this thermomechanical treatment was further machined to bars (5 mm by 1 mm by 1 mm) for further in situ SXRD and DIC experiments.

### E-DE BFH sample preparation

The normal FE BF-BT ceramics were first machined to bar (5 mm by 1 mm by 1 mm)–shaped samples, followed by a poling step along the length direction. The poling procedures were performed at 80°C with an electric field of 5 MV m^−1^ for 10 min. The poled samples were then re-electroded on the parallel rectangular surfaces using a silver paste (GWENT C2130823D1) as shown in Fig. 1F. Thermal aging of the poled samples was performed at 475°C for 3 and 4 hours with the same heating and cooling rate of 10°C min^−1^ to achieve internal bias fields about 1.5 and 1.75 MV m^−1^, respectively.

### Fracture toughness determination by the SEVNB method

The fracture toughness of AS, normal FE and tempered normal FE samples (*K*_IC_) were determined by the single-edge V-notched beam (SEVNB) method following the ASTM standard (ASTM C1421-18). Rectangular-ended beam specimens [12 mm by 2 mm (*W*) by 1.5 mm (*B*)] were cut from the well-sintered pellets using precision diamond wheel cutting to satisfy the configuration requirements for the SEVNB test. An ultrasharp V-notch was subsequently introduced with depth along the height direction of test specimens performed with Helios 5 Laser-PFIB equipped with fully integrated femtosecond laser. At least five valid beams were tested for each sample with different heat treatment conditions. The fracture toughness was evaluated from the fracture load and specimen geometries, as follows (*48*)

$$K_{\text{IC}}=g\left(\frac{P_{\text{max}}S_{0}10^{-6}}{BW^{\frac{3}{2}}}\right)\left(\frac{3\times {\left(\frac{a}{W}\right)}^{\frac{1}{2}}}{2\times {\left(1-\frac{a}{W}\right)}^{\frac{3}{2}}}\right)$$ (1)

where *g* is a dimensionless correction factor given as

$$g=g\left(\frac{a}{W}\right)=\frac{1.99-\left(\frac{a}{W}\right)\left(1-\frac{a}{W}\right)\left[2.15-3.93\left(\frac{a}{W}\right)+2.7{\left(\frac{a}{W}\right)}^{2}\right]}{1+2\left(\frac{a}{W}\right)}$$ (2)

where *P*_max_ is the fracture load. *a* is the depth of premade notch. *B*, *W*, and *S*_0_ refer to the sample height (2 mm), width (1.5 mm), and span width (10 mm), respectively.

### Ferroelectric, piezoelectric, and dielectric property measurements

Ferroelectric *P*-*E* hysteresis loop measurements were performed on the BF-BT ceramics with a high voltage sinusoidal waveform at a frequency of 1 Hz, using an HP33120A function generator in conjunction with a Chevin Research HVA1B amplifier. The dielectric permittivity was measured upon heating from room temperature to 700°C with a heating rate of 2°C/min and at frequencies of 1, 10, and 100 kHz, using an HP 4284A precision LCR meter. The samples were poled under a dc field of 6 kV/mm at 100°C for 10 min. The ex situ temperature dependence of piezoelectric coefficient *d*_33_ was determined by quasistatic measurements of the *d*_33_ values at room temperature with a Sinocera YE2730A *d*_33_ meter after the samples were stabilized at multiple specified temperatures (25° to 850°C) for 30 min followed by air quenching to room temperature. The temperature-dependent piezoelectric coefficient (*d*_33_) and coupling factor (*k*_33_) was measured by fitting the impedance spectra with the approach proposed by Sherrit *et al.* (*49*) to mitigate the high dielectric loss of the piezoelectrics at elevated temperatures. All the material parameters are considered as complex forms, and the loss is given by the corresponding imaginary part. The implementation of the impedance spectrum fitting was performed with an in-house developed MATLAB script.

### S/TEM sample preparation and imaging methods

S/TEM samples were prepared using the FEI Helios Nanolab 660 focused ion beam (FIB) scanning electron microscope for thinning to produce a cross-sectional lamella. The lamella sample was prepared using gradually decreasing ion beam milling voltages of 30 16, and 8 kV for thinning, followed by, 5, 2, and 1 kV for polishing (*50*). Afterward, the TEM samples were transferred to the Precision Ion Polishing System II (PIPS II) for the final polishing to eliminate the damage layer induced by FIB. Final polishing was done with an ion beam voltage of 0.3 and 0.1 kV at cryogenic temperature. This refined TEM sample preparation process plays a key role in revealing the fine spinodal decomposition patterns by removing artifacts that can be present due to FIB damage. The lamellae were prepared to be thin (less than 30 nm, confirmed by EELS). An additional needle-shaped sample for TEM tomography was prepared by FIB using the same parameters mentioned above.

TEM imaging was performed on a Talos F200X (Thermo Fisher Scientific) operated at 200 kV. STEM imaging and analysis techniques were performed on the Titan G2 80-200 S/TEM ChemiSTEM (Thermo Fisher Scientific) and double-corrected JEOL ARM300F (ePSIC, Diamond Light Source, UK) microscopes operating at 200 and 300 kV, respectively. HAADF images were collected in the range of 54 to 200 mrad with a probe forming a convergence semiangle of 21 mrad. The influences of the sample thickness, sample tilting, and contamination diffraction contrast were carefully minimized by the preparation of thin FIB samples (less than 30 nm with a local thickness variation less than 1 nm for the region of interests, confirmed by EELS) and accurate electron beam alignment along the zone axis to avoid parasitic angular deviation of intensity distribution within diffracted beam. For accurate atom position and displacement measurements, each atomic-resolution image is reconstructed from 20 frames (2048x2048) of orthogonal fast scans, which were aligned using SmartAlign for rigid and nonrigid distortions correction. Atom column positions were determined using atom column indexing (*51*) and Atomap (*52*) with further polarization distribution analysis being performed using an in-house MATLAB script. Geometric phase analysis (GPA) was performed with Strain++ (*53*) using a mask size roughly equal to half the reciprocal lattice parameter. The STEM-EDX spectrum imaging was acquired using the Titan’s SuperX EDX detectors and processed within the Velox software. STEM-EELS was performed using the Titan’s GIF Quantum ER System with an entrance aperture of 5 mm, 0.1-s total dwell time, and a dispersion of 0.25 eV per channel.

The HAADF-STEM image series for tomographic construction was acquired with a Fischione 2020 single tilt tomography holder ranging from ±70° with a 5° interval. Tomographic reconstruction was performed using the SIRT algorithm with the Etomo package. Further segmentation and visualization of the tomographic data were performed using the Thermo Fisher Scientific Avizo package.

### Phase-field modeling

A bulk perovskite single crystal was modeled with a conserved composition field c(r,t)∈[0.6,0.8] (A-site Bi fraction) and a nonconserved polarization vector $P=\left(P_{x}+P_{y}+P_{z}\right)$. The total free energy density is

$$\begin{array}{c} f=f_{\text{Landau}}\left(P;c,T\right)+f_{\text{grad}}\left(\nabla P\right)\\ +f_{\text{CH}}\left(c,\nabla c\right)+f_{\text{elastic}}\left(P,c\right)+f_{\text{electrostatic}}\left(P,c,\phi \right) \end{array}$$ (3)

where $f_{\text{Landau}}$ is a composition-dependent and temperature-dependent Landau polynomial for PZT-like endmembers mapped to BF-BT via x=1−c, $f_{\text{grad}}$ is a polarization gradient energy with *G*-tensor ($G_{11},G_{44},G_{44}^{\prime }$), $f_{\text{CH}}$ is the Cahn-Hilliard term of the total free energy governs phase separation (spinodal decomposition) and interfacial energy between compositionally distinct regions, $f_{\text{elastic}}$ includes periodic microelasticity with Vegard (hydrostatic) and electrostrictive eigenstrains, and $f_{\text{electrostatic}}$ is electrostatic energy with short-circuit boundary conditions. Detailed description of each energy term can be found in the Supplementary Materials.

All simulations were carried out in a 3D periodic cell of size 256 by 256 by 256 grid points. The grid spacing was chosen as $d_{x}=d_{y}=d_{z}=0.8\;\text{nm}$, giving a simulation box of ∼205 nm. This high spatial resolution was needed to resolve domain walls and compositional gradients on the order of 1 to 5 nm. All operators are evaluated spectrally on a periodic 3D grid using FFTs. The computations were accelerated using GPU-based FFT solvers and an optimized phase-field code (in MATLAB) to handle the large grid. We follow the experimental thermal histories and poling routes used to create BFH microstructures for phase-field modeling, which includes initialization, prepoling, zero-field relax, thermal aging, post-aging relax, and property evaluation. The details of each step are included in the Supplementary Materials. The estimation of local chemical free energy, Landau, elastic, and electrostatic parameters can also be found in the Supplementary Materials.

### In situ SXRD measurements

In situ SXRD experiments were performed at beamline I15 (Diamond Light Source, UK) with a photon energy of 72 keV, a beam diameter of 76 μm by 115 μm (vertical x horizontal) and using transmission geometry. The direction of the applied electric field (ψ = 0°) is parallel with the axial direction (*X*_3_; shown in Fig. 2H) of the M-DE BFH sample and transverse direction (*X*_1_; shown in Fig. 3A) of the E-DE BFH sample, respectively. 2D diffraction patterns were caked into 24 banks (15° per bank) along azimuthal angles ψ from 0° to 360° and integrated into 24 corresponding 1D line profiles. Further details of the experimental setup, data collection and postprocessing are provided in our previous study (*54*).

The Daymond method (*54*) was adopted to estimate the macroscopic strain at a given azimuthal angle, $S_{m}\left(\psi \right)$, taking account of the effective lattice strains for the representative grain orientations {222}, {200}, and {220}, given as

$$S_{m}\left(\psi \right)=\frac{\sum m_{\left\{\mathrm{hkl}\right\}}S_{\left\{\mathrm{hkl}\right\}}^{*}\left(\psi \right)}{\sum m_{\left\{\mathrm{hkl}\right\}}}$$ (4)

where $m_{\left\{\mathrm{hkl}\right\}}$ is the total multiplicity of grain family {*hkl*} for {222}, {200}, and {220}, respectively. The effective lattice strain for each grain family $S_{\left\{\mathrm{hkl}\right\}}^{*}\left(\psi \right)$ is determined by the effective *d*-spacing $d_{\left\{\mathrm{hkl}\right\}}^{*}\left(\psi \right)$, and $d_{\left\{\mathrm{hkl}\right\}}^{*}\left(\psi \right)$ is obtained by a weighted average process, taking account of the domain fraction $\eta_{\left(\mathrm{hkl}\right)}\left(\psi \right)$ and *d*-spacing of the reflection (*hkl*), $d_{\left(\mathrm{hkl}\right)}\left(\psi \right)$, which can be respectively expressed as

$$S_{\left\{\mathrm{hkl}\right\}}^{*}\left(\psi \right)=\frac{d_{\left\{\mathrm{hkl}\right\}}^{*}\left(\psi \right)-d_{\left\{\mathrm{hkl}\right\}}^{0}\left(\psi \right)}{d_{\left\{\mathrm{hkl}\right\}}^{0}\left(\psi \right)}$$ (5)

$$d_{\left\{\mathrm{hkl}\right\}}^{*}\left(\psi \right)=\sum \eta_{\left(\mathrm{hkl}\right)}\left(\psi \right)d_{\left(\mathrm{hkl}\right)}\left(\psi \right)$$ (6)

where $d_{\left\{\mathrm{hkl}\right\}}^{0}\left(\psi \right)$ is the initial effective lattice spacing at a given azimuthal angle, which acts as the relative zero strain lattice spacing at each bank in comparison with the strain free lattice spacing before domain engineering. The analysis methods used for detailed evaluation of the domain fractions of different grain families, $\eta_{\left(\mathrm{hkl}\right)}\left(\psi \right)$, are given in our previous study (*54*). The estimated macroscopic axial and transverse strains for M-DE BFH ceramics are achieved at ψ = 0 and 90° for $S_{m}\left(\psi \right)$. The estimated macroscopic engineering shear strain, *S*_5_, for E-DE BFH ceramics is double the magnitude of the tensorial shear strain, *S*_31_, which is achieved at ψ = 45° for $S_{m}\left(\psi \right)$. By considering the 90° rotation of the global axes and the deviation of principle strain away from the global axes in shear mode, the orientation distribution of macroscopic strain, $S_{m}\left(\psi \right)$, in E-DE BFH ceramics follows a modified second-order spherical harmonic function (*54*), given as

$$S_{m}\left(\psi \right)=\left(d_{33}^{\prime }-d_{31}^{\prime }\right)E{\text{cos}}^{2}\left(\psi -∆\psi \right)+d_{31}^{\prime }E$$ (7)

where ∆ψ is the principal strain angle, $d_{31}^{\prime }$ and $d_{33}^{\prime }$ are the effective piezoelectric coefficients parallel and perpendicular to the principal strain direction, respectively, and *E* is the strength of electric field. Therefore, the principal strain angle ∆ψ can be determined by nonlinear fitting of the experimental data of $S_{m}\left(\psi \right)$ with Eq. 4. The associated XRD peak profile fitting and the evaluation of DOD and SOD were performed with in-house developed MATLAB scripts.

### DIC strain measurements

The macroscopic strain–electric field (*S*-*E*) loops of E-DE and M-DE BFH samples were measured with a 2D DIC method under an optical microscope. A square-ended bar specimen was placed in a custom-designed sample holder with electrical contact to the HV amplifier. The images for strain calculation were acquired from the cross section of the specimen immersed in silicone oil to avoid electrical arcing in air. A triangular electric field waveform with a frequency of 0.1 Hz was applied for all the measurements for compatibility with the image acquisition rate of 8 fps. The image acquisition, electric field application and recording were controlled by a LabVIEW program. The sequence of images was processed with the commercial DIC software (LaVision Davis 10.2.1) to determine the 2D deformation relative to the initial zero-field reference image. Further details of the DIC experiment setup and strain calculation can be found in our previous study (*55*).

## Supplementary Materials

### The PDF file includes:

Figs. S1 to S15 Legends for movies S1 to S5 Supplementary Text References
