# Supplementary material for: Bulk ferroelectric heterostructures
Source: Sci Adv. 2026 Oct 2;12(40):eaef9861. doi: 10.1126/sciadv.aef9861 (PMC13632740; doi:10.1126/sciadv.aef9861)
Supplement: Supplementary file 1 [file sciadv.aef9861_sm.pdf]

Science Advances
AAAS

Supplementary Materials for

Bulk ferroelectric heterostructures

Yizhe Li *et al.*

Corresponding author: Yizhe Li, yizhe.li@manchester.ac.uk; David A. Hall, david.a.hall@manchester.ac.uk

Sci. Adv. **12**, eaef9861 (2026)
DOI: 10.1126/sciadv.aef9861

The PDF file includes:

Figs. S1 to S15
Legends for movies S1 to S5
Supplementary Text
References

Other Supplementary Material for this manuscript includes the following:

Movies S1 to S5

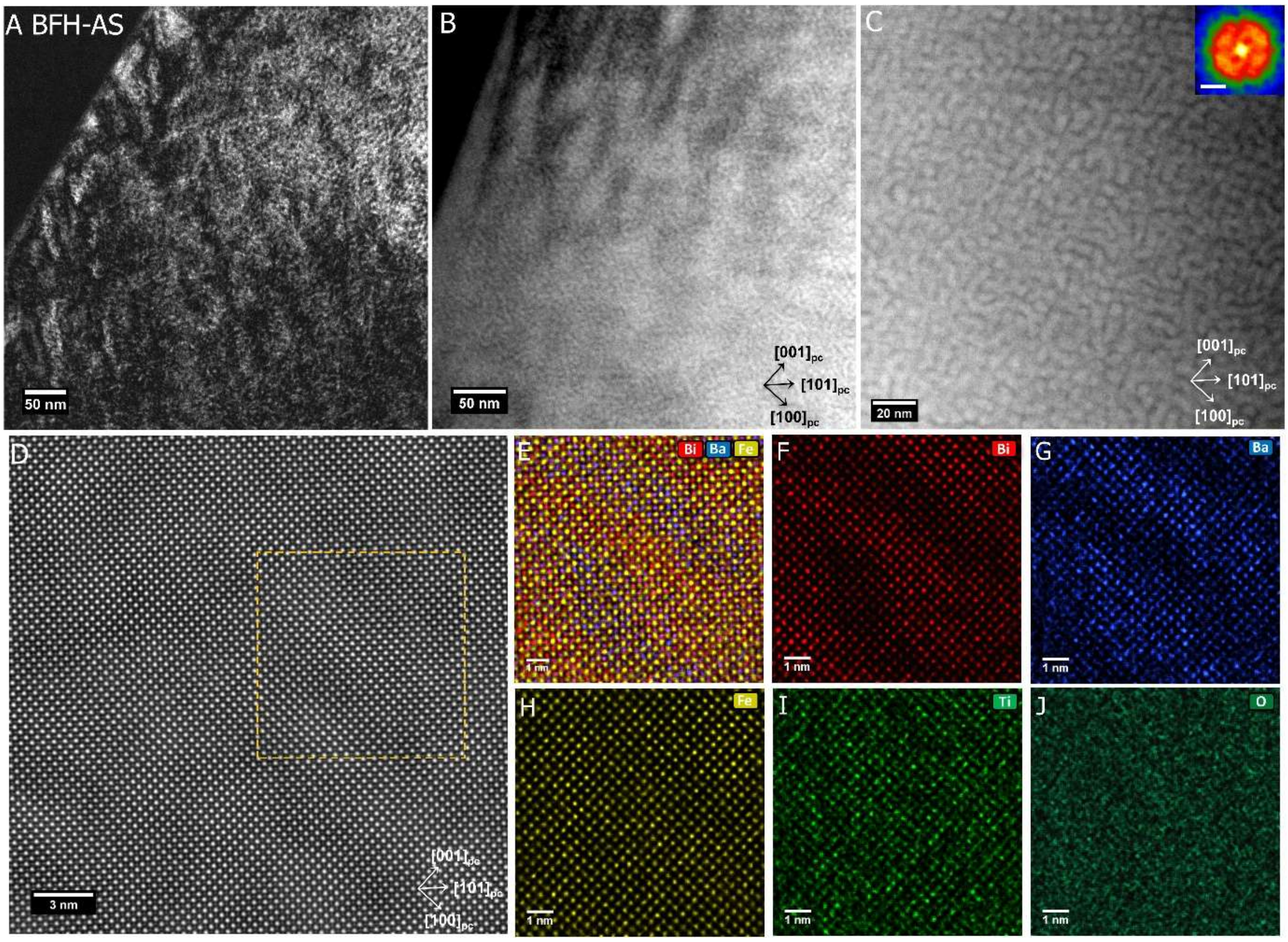

Fig. S1 **Observation of nanoscale elemental segregation induced by thermal ageing of BFH-AS 0.7BiFeO$_3$-0.3BaTiO$_3$ ceramic**. **A**, Dark field TEM image of [010] orientated grain in BFH(AS) 0.7BiFeO$_3$-0.3BaTiO$_3$ ceramics, showing labyrinth domains with local nano features. **B**, HAADF-STEM investigation of the nanoscale domain structure induced by thermal ageing. **C**, HAADF-STEM image of an enlarged region from **B**, showing combined stripe and cuboidal features with slightly preferred orientation along [001] and [100] directions with the 4-fold side lobes in the FFT of **C** in the inset. **D**, atomic resolution HAADF-STEM image of nanoscale features showing coherent lattice structure. **E-J**, atomic EDS elemental mapping of Bi, Ba, Fe, Ti and O for the identical region (dashed square) marked in **D**. The scale bars are 0.2 nm^{-1} for the inset in **C. C** reproduces the HAADF-STEM image shown in Fig. 1 **C**.

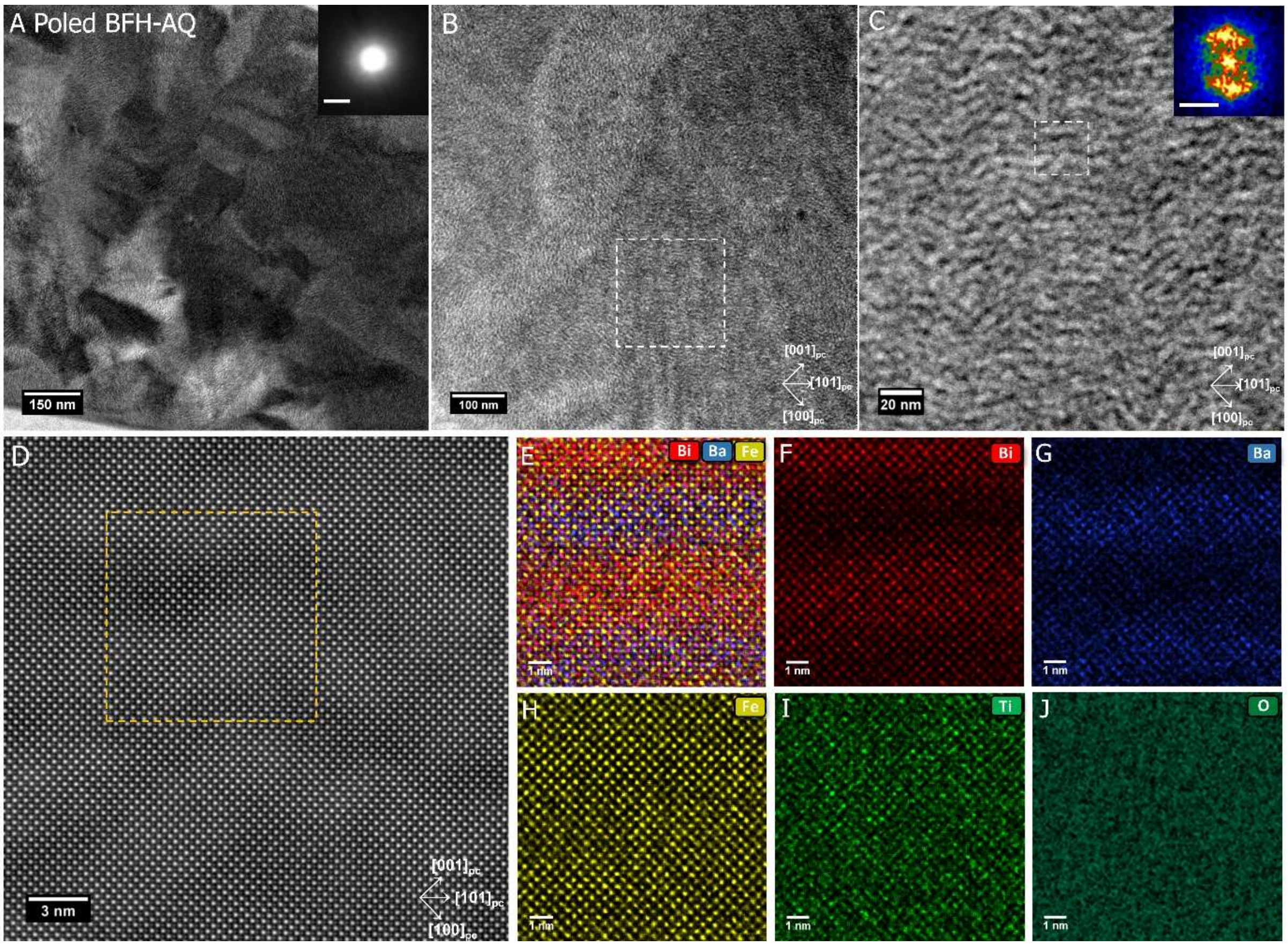

Fig. S2 **Observations of nanoscale elemental segregation in poled BFH-AQ 0.7BF-0.3BT ceramic**. **A**, Bright field TEM image of $[010]_{pc}$ orientated grain in poled BFH-AQ 0.7BF-0.3BT ceramics, showing nanoscale wave-like patterns in each individual domain. The inset shows the central diffraction spot with spinodal ring from the selected region in **A**. **B**, HAADF-STEM investigation of the phase-separated regions within different parental ferroelectric domains induced by thermal ageing. **C**, HAADF-STEM image of enlarged region (dashed square) from an individual parent domain in **B**, showing orientated growth of nanoscale stripes along $[101]_{pc}$ direction with 2-fold side lobes in the FFT of **C** in the inset. **D**, atomic resolution HAADF-STEM image showing coherent lattice structure extending across the nanoscale regions (dashed square in **C**). **E-J**, atomic EDS elemental mapping of Bi, Ba, Fe, Ti and O for the identical region (dashed square) marked in **D**. The scale bars are 0.2 nm^{-1} for the insets in **A** and **C**. **C** reproduces the HAADF-STEM image shown in Fig. 1 **E** and Fig. 2 **E**.

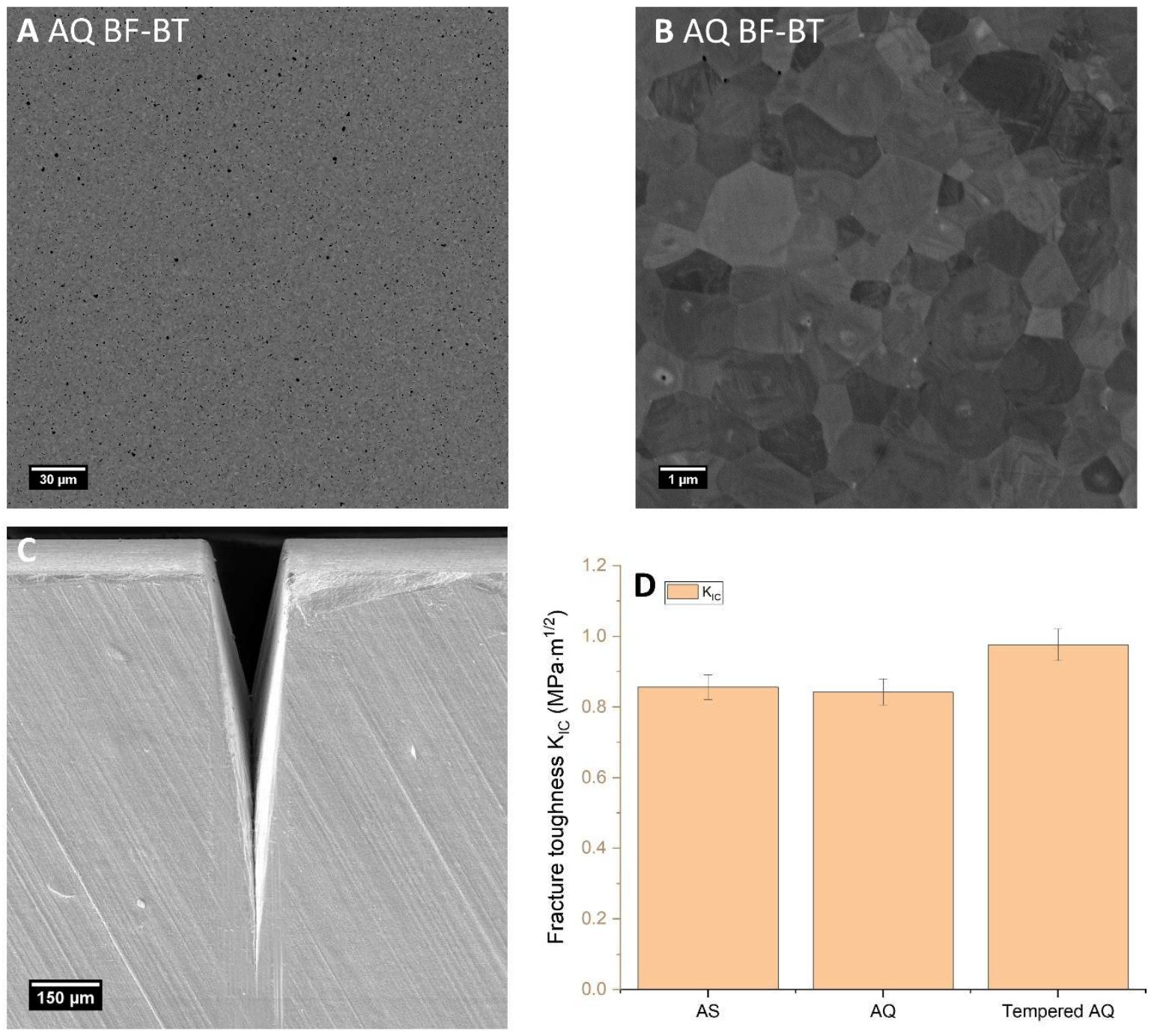

Fig. S3 **Microstructure and fracture toughness of BF–BT ceramics. A-B**, SEM micrographs of polished cross section of normal FE BF-BT sample (air quenched) showing features free of microcracks. **C**, SEM micrograph of V-shaped notch prepared with laser PFIB on normal FE BF-BT sample. **D**, the fracture toughness of as sintered (AS), air quenched (AQ) and tempered AQ 0.7BF-0.3BT ceramics.

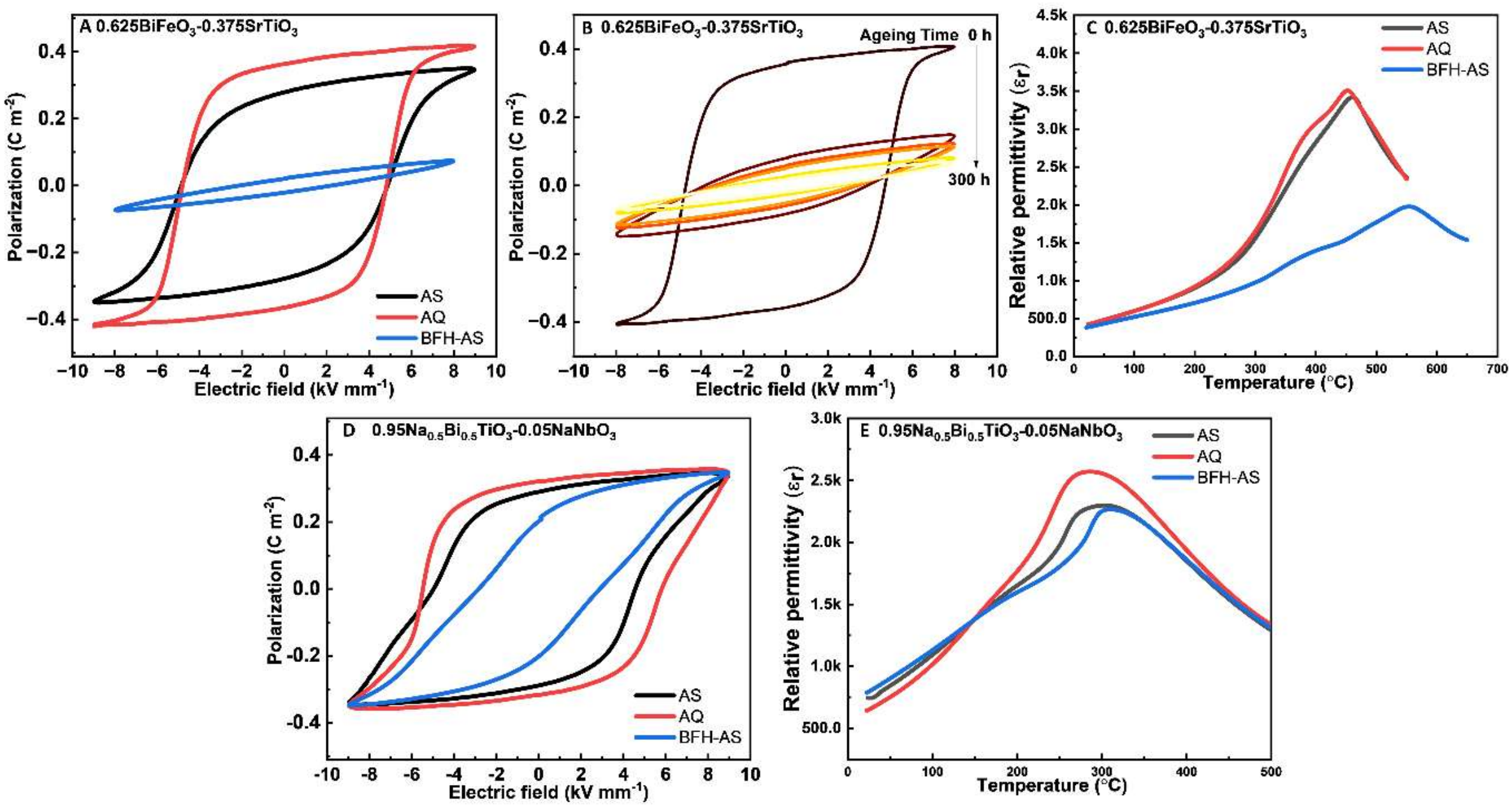

Fig. S4 **Exploitation of the thermal treatment approach on the 0.625BiFeO$_3$-0.375SrTiO$_3$ (0.625BF-0.375ST) and 0.95Na$_{0.5}$Bi$_{0.5}$TiO$_3$-0.5NaNbO$_3$ (0.95NBT-0.5NN) solid solutions. A**, Polarisation-electric field (P-E) hysteresis loops of as sintered (AS), air quenched (AQ) and 300 hour thermal aged BFH-AS 0.625BF-0.375ST ceramics. **B**, Impact of thermal ageing on P-E loops of AQ 0.625BF-0.375ST ceramic as a function of dwelling time at 500 °C. **C**, temperature dependence of relative permittivity for as sintered (AS), air quenched (AQ) and thermally aged (500 °C for 300 h) BFH(AS) 0.625BF-0.375ST ceramics. **D**, Polarisation-electric field (P-E) hysteresis loops of as sintered (AS), air quenched(AQ) and thermal aged (700 °C for 100 h) BFH-AS 0.95NBT-0.5NN ceramics. **E**, temperature dependence of relative permittivity for as sintered (AS), air quenched (AQ) and thermally aged (700 °C for 100 h) BFH-AS 0.95NBT-0.5NN ceramics.

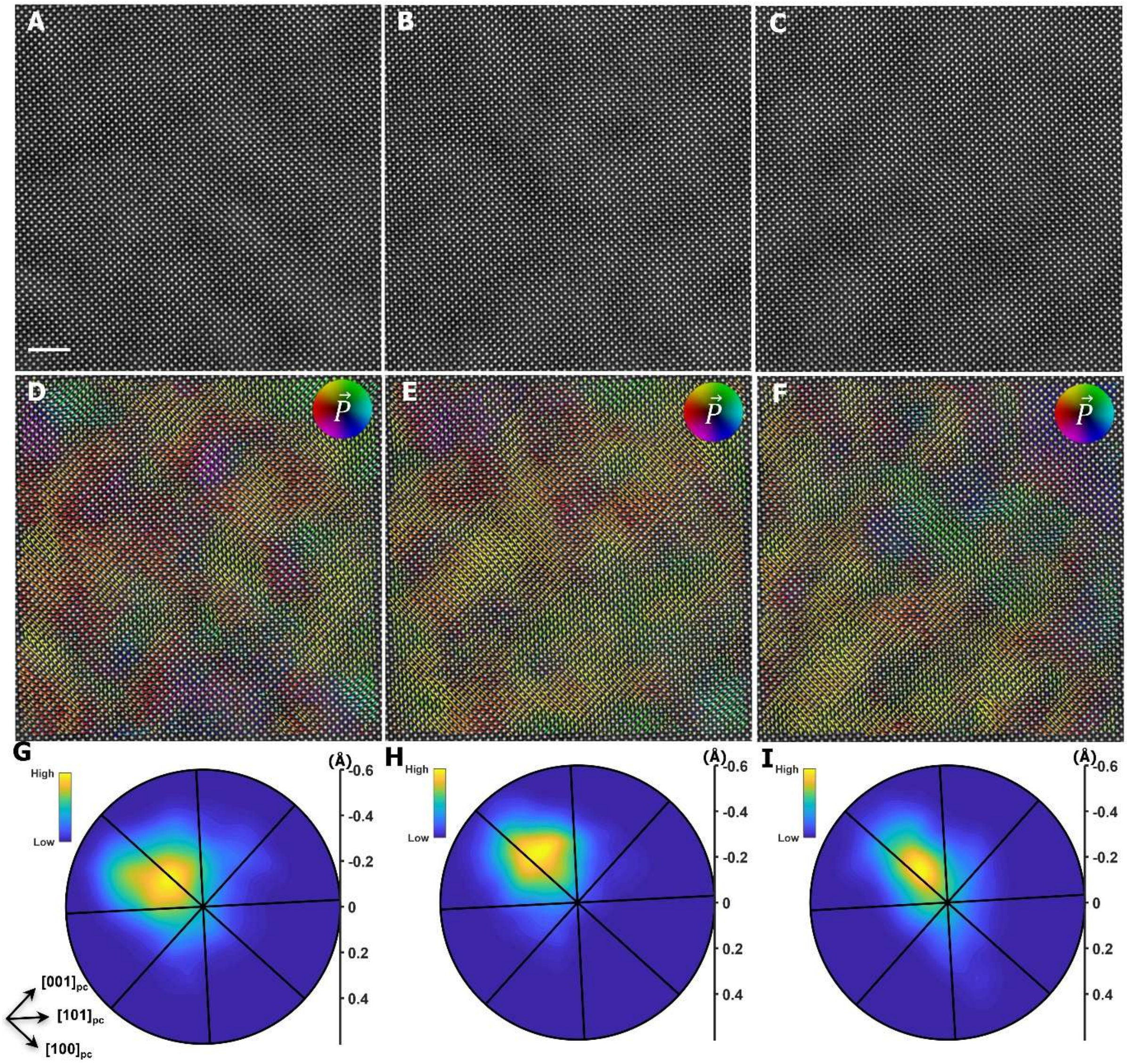

Fig. S5 **Polarisation orientation distribution of nanoscale features in BFH-AS 0.7BiFeO$_3$-0.3BaTiO$_3$ sample. A-C**, Atomic resolution HAADF images of exemplar regions of nanoscale features along [010] zone axis, label bar indicates a length of 3 nm. **D-F**, Atomic scale polarisation vector mapping of identical regions of **A-C** respectively. **G-I** Polar plot of polarisation vector orientation distribution for the same region of **A-C respectively**.

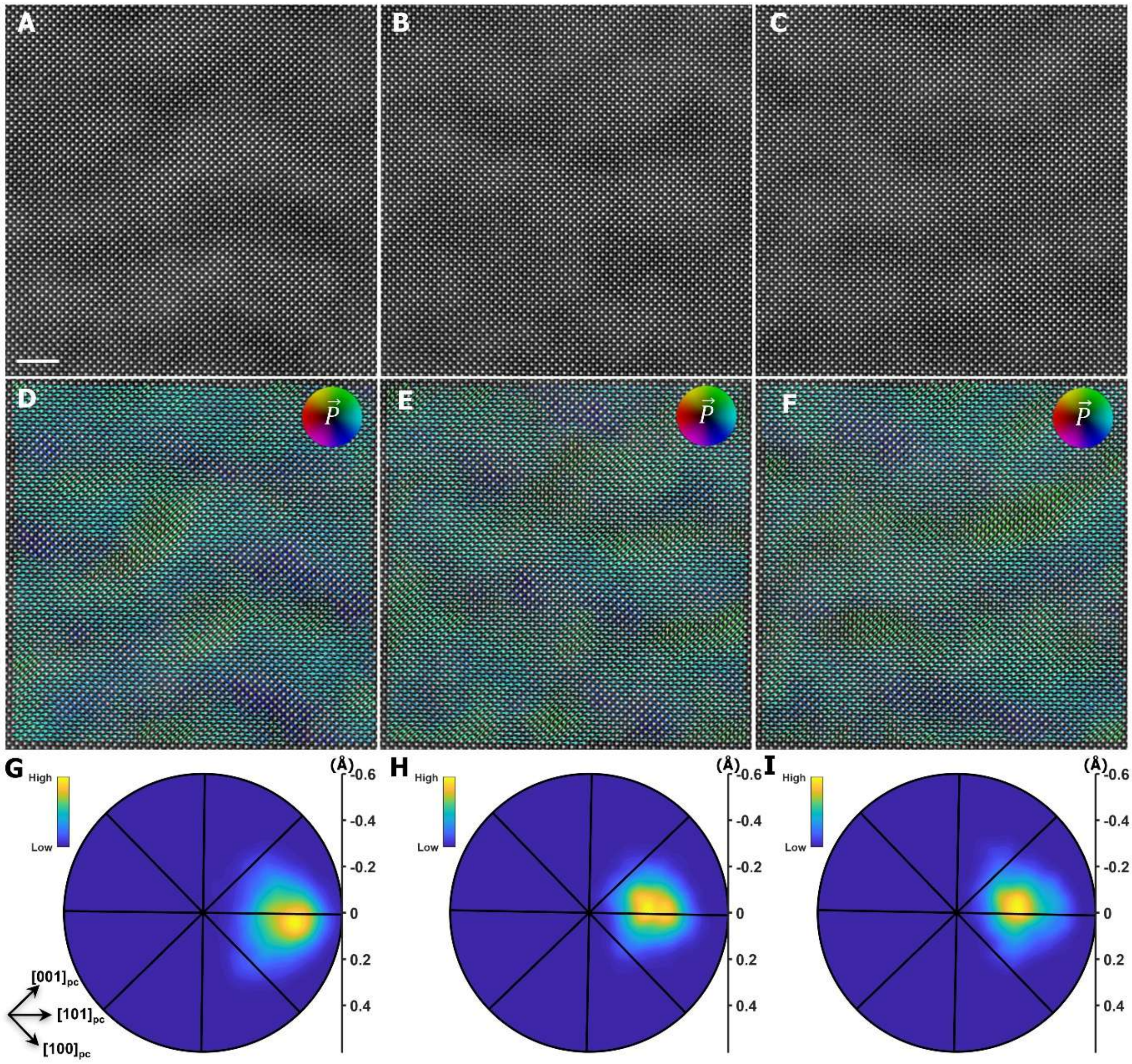

Fig. S6 **Polarisation orientation distribution of nanoscale wave-like structure of poled BFH-AQ 0.7BiFeO$_3$-0.3BaTiO$_3$ sample. A-C**, Atomic resolution HAADF images of exemplar regions of nanoscale wave-like structure along [010] zone axis, label bar indicates a length of 3 nm. **D-F**, Atomic scale polarisation vector mapping of identical regions of **A-C** respectively. **G-I** Polar plot of polarisation vector orientation distribution for the same region of **a-c respectively**.

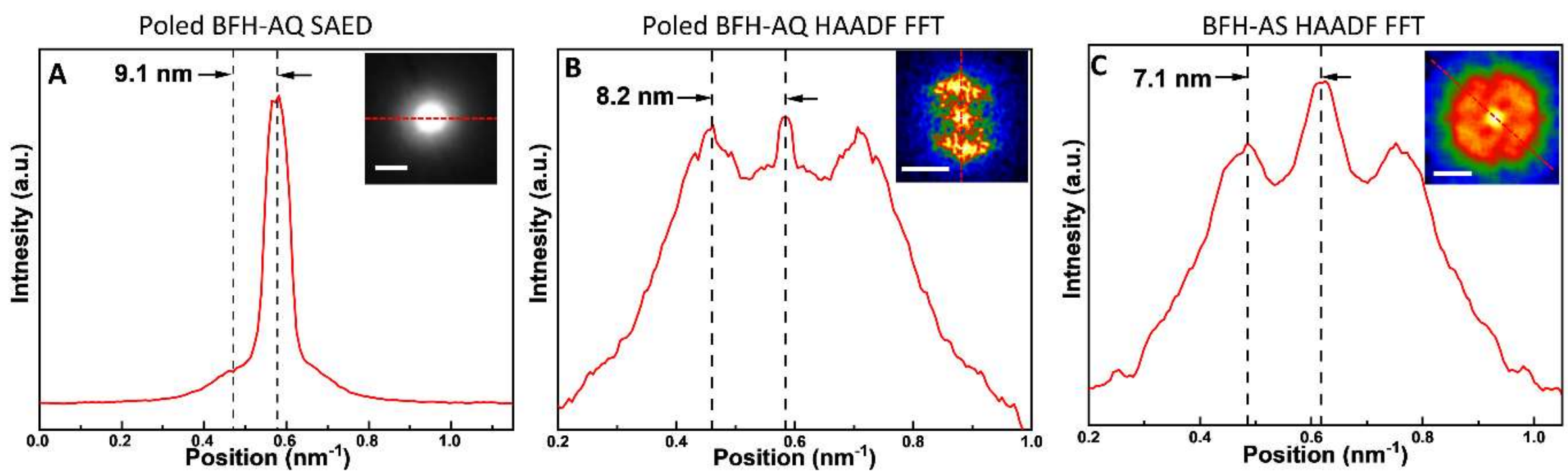

Fig. S7 **Comparing scale of compositional modulation according to SAED and FFT of HAADF images**. The scale bars are 0.2 nm^{-1} for each of the insets in **A**, **B** and **C**

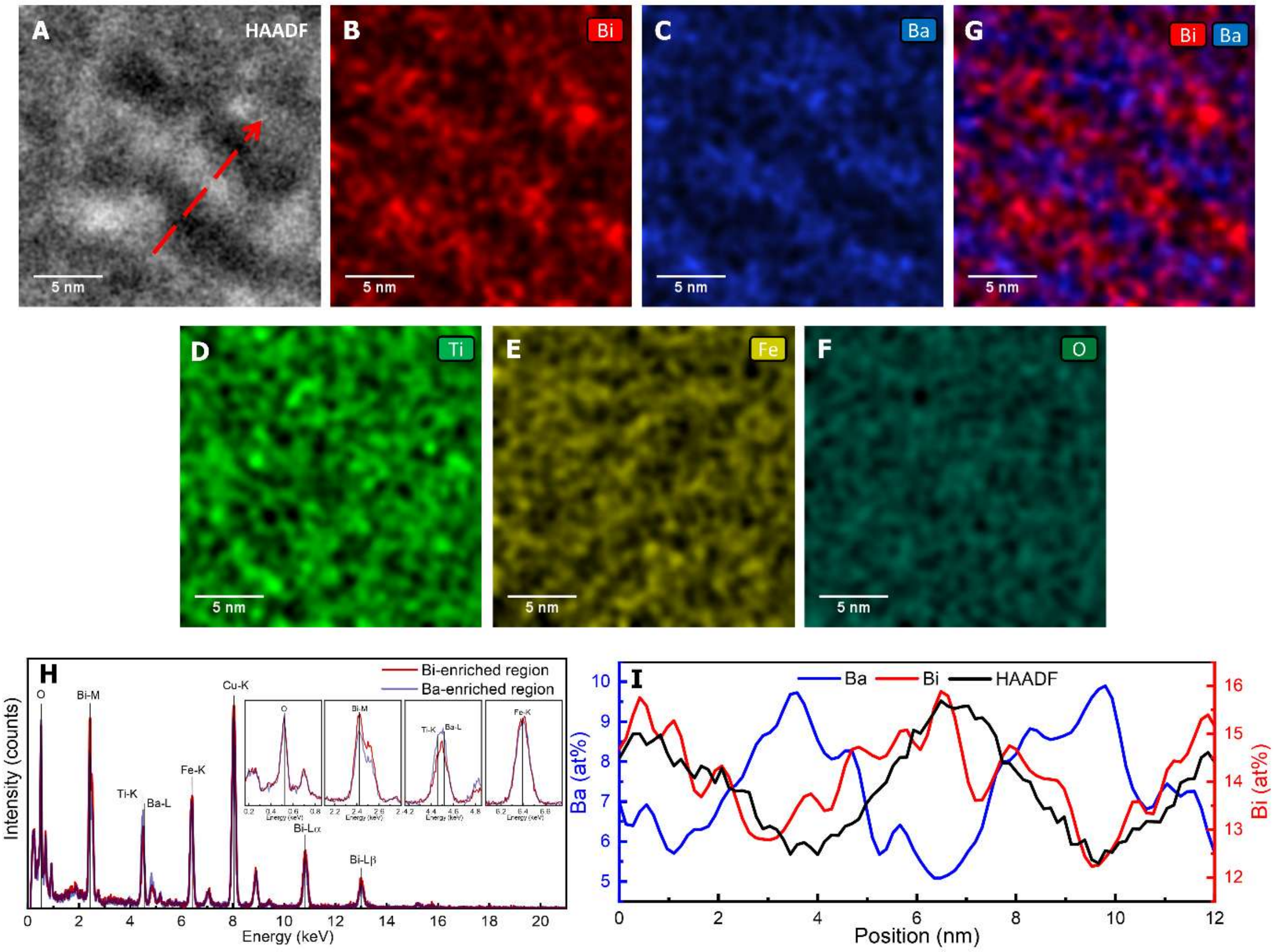

Fig. S8 **Off zone axis EDS mapping of the phase-separated regions in poled BFH-AQ 0.7BiFeO$_3$-0.3BaTiO$_3$ samples.**

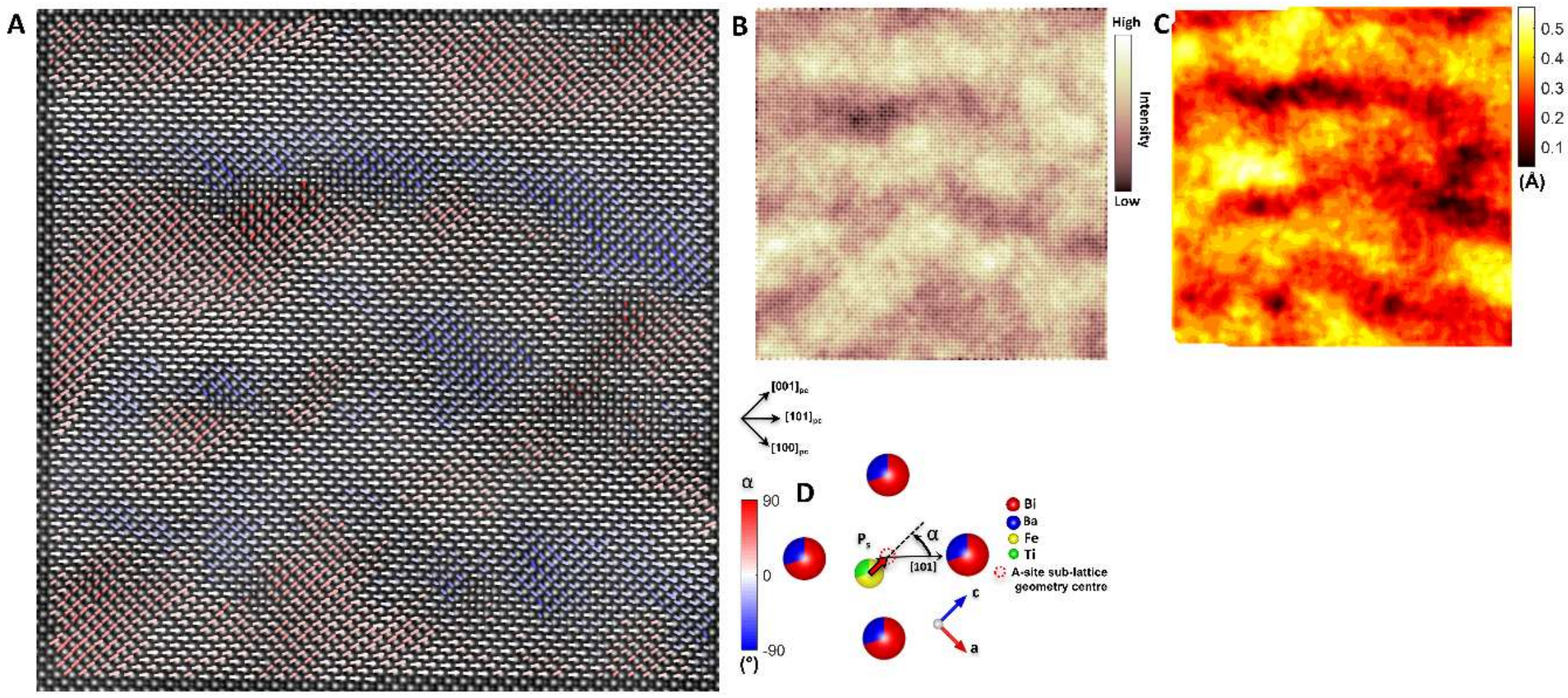

Fig. S9 **Atomic-scale mapping of local polarization vectors and B-site cation displacements**. **A**, Atomic scale polarisation vector mapping with colour indexing local polarisation vectors by the angle, α, relative to the $[101]_{pc}$ direction. **B,** Intensity distribution and **C,** mapping of the B-site cation displacement of the same area in **A**. **D**, Schematic illustration of the misorientation angle, α, between the local polarisation vector and the $[101]_{pc}$ direction.

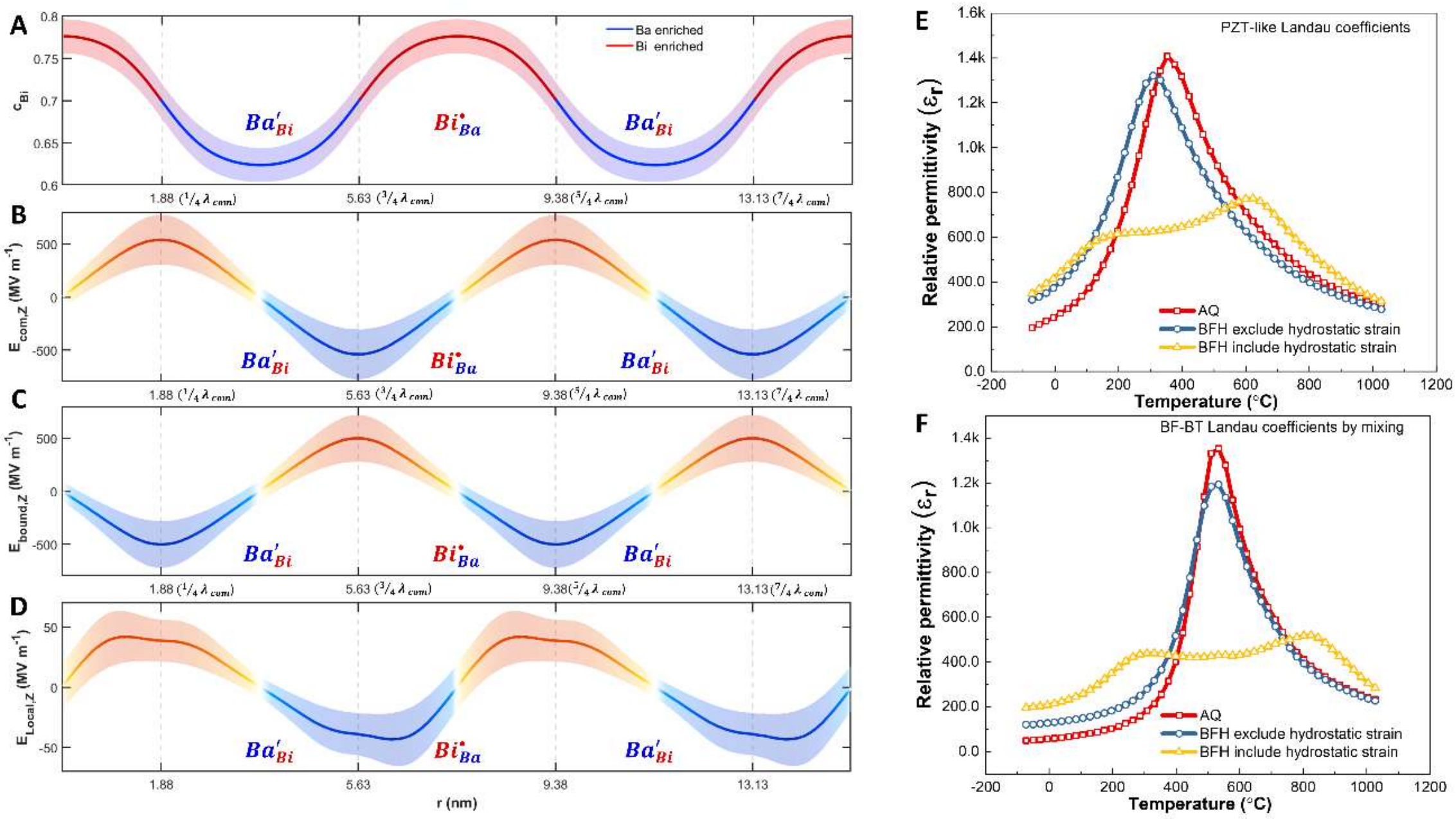

Fig. S10 **Phase-field simulation of composition-modulated electric fields and temperature-dependent dielectric response**. The variations of Bi^{3+} concentration (c_{Bi}), the component of electric field induced by Bi concentration variation along Z direction ($E_{com,Z}$), the component of electric field induced by polarisation bound charge along Z direction ($E_{bound,Z}$), the component of local electric field along Z direction ($E_{Local,Z} = E_{com,Z} + E_{bound,Z}$) over the composition wave, λ_{com}, for 2 periods, obtained by phase binning the whole simulated phase field model and shown in **A**-**D** respectively. **E**&**F**, the temperature dependent relative permittivity along [111] obtained by phase field modelling for air quenched normal BF-BT ferroelectrics, and BFHs excluding or including the contribution of hydrostatic strain, using PZT-like or BF-BT Landau coefficients respectively.

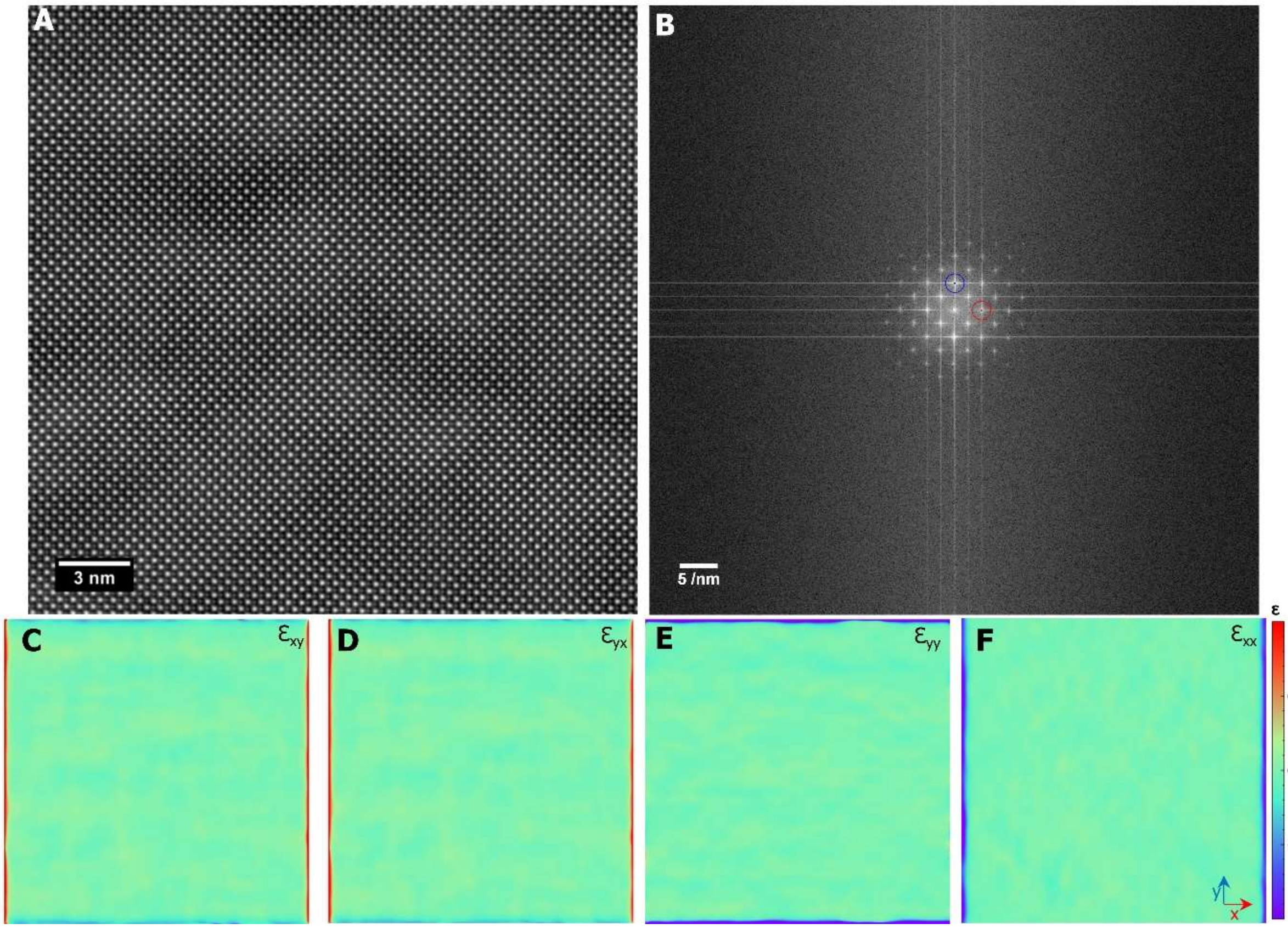

Fig. S11 **Geometry phase analysis (GPA) for strain mapping of poled BFH-AQ 0.7BiFeO$_3$-0.3BaTiO$_3$ ceramic.** The strain mappings (**C-F**) of the HAADF image of region **A** show negligible lattice strain, which indicates that the regions showing elemental segregation are under a hydrostatic stress status. The reflections used for GPA are highlighted by blue and red circles in **B**. Panel **A** reproduces the HAADF-STEM image shown in Fig. S2 D, and is included here as the source image for the GPA strain analysis.

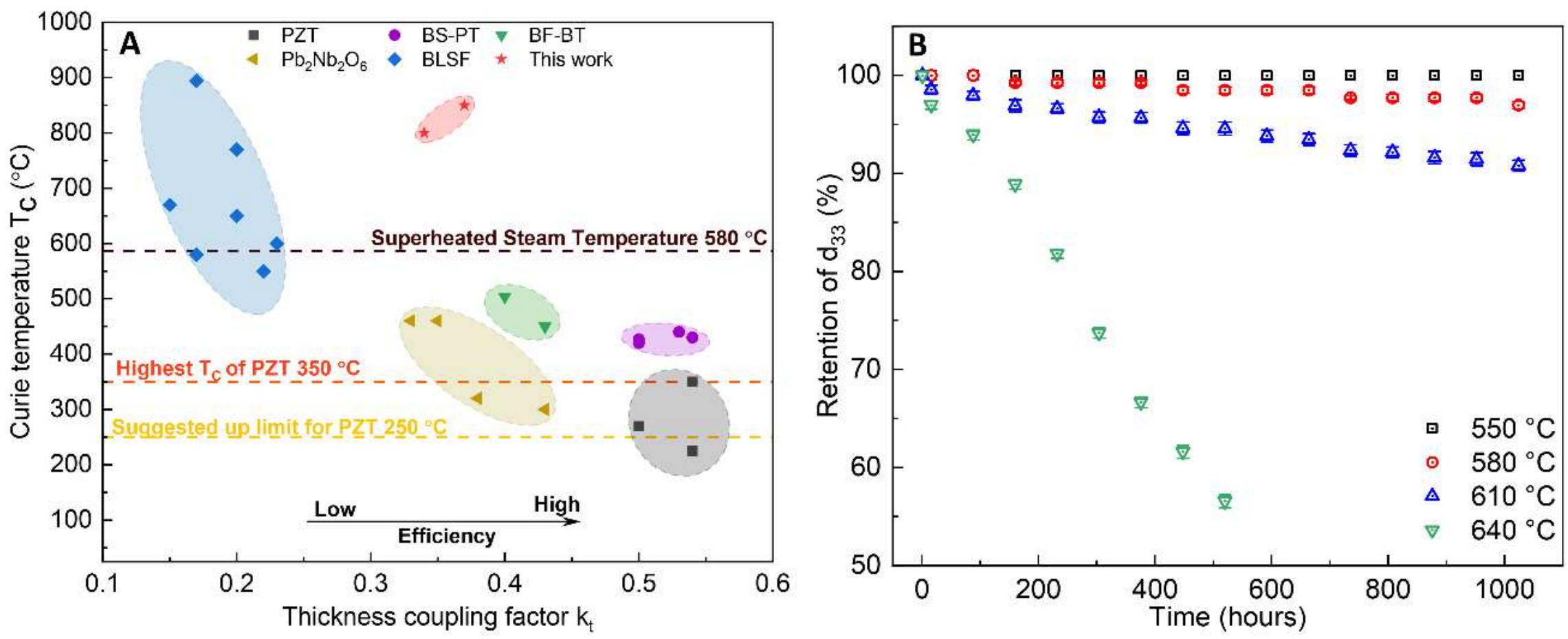

Fig. S12 **Electrical properties and associated stability of 0.7BF-0.3BT BFH ceramics.** Comparison of **A**, thickness coupling factor (k_t) between the BFH ceramics and other perovskite and non-perovskite ceramics, including PbZr$_x$Ti$_{(1-x)}$ (PZT), BiScO$_3$-PbTiO$_3$ (BS-PT), BF-BT, Pb$_2$Nb$_2$O$_6$ and bismuth layer-structured ferroelectrics (BLSF). **B,** retention of d_{33} after dwelling at various temperatures for poled BFH samples with different durations up to 1000 hours.

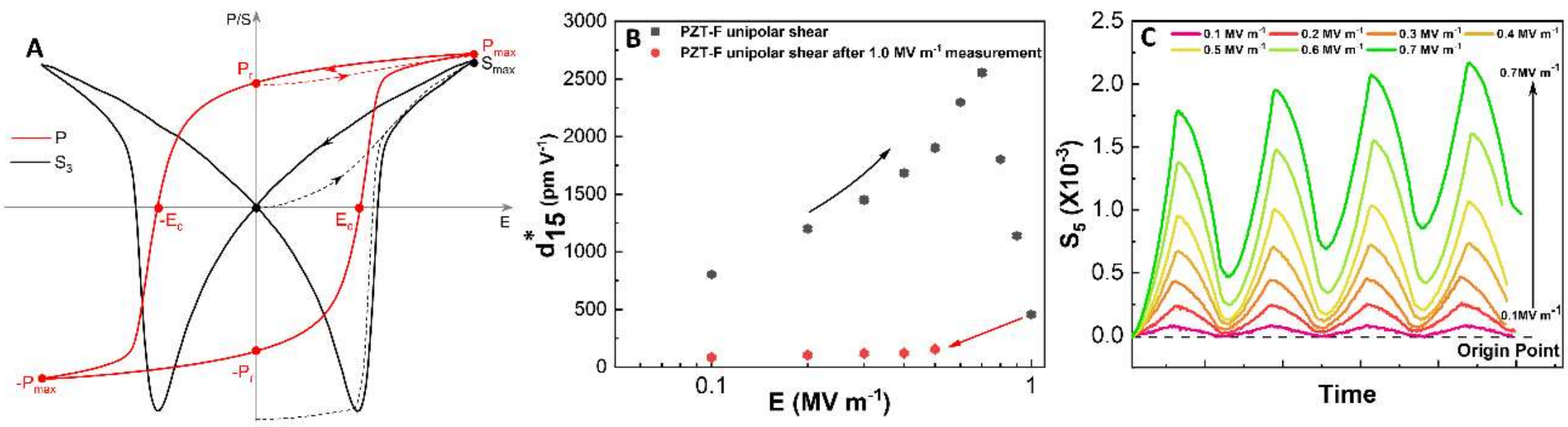

Fig. S13 **Polarisation hysteresis and cross-poling of normal ferroelectrics**. **A**, The illustration of polarisation and strain hysteresis behaviours in normal ferroelectrics under both unipolar and bipolar electric fields. **B**, effective shear piezoelectric coefficient, d_{15}^{*}, of soft PZT-F ceramics measured at various electric field levels up to 1 MV m^{-1} and the repetition of the measurements after the exposure to 1 MV m^{-1}. **C**, shear electrostrain behaviour, S_5, of PZT-F ceramics under 4 consecutive cycles of unipolar field at different levels, showing significant shift in origin point and gradual depoling at high field levels.

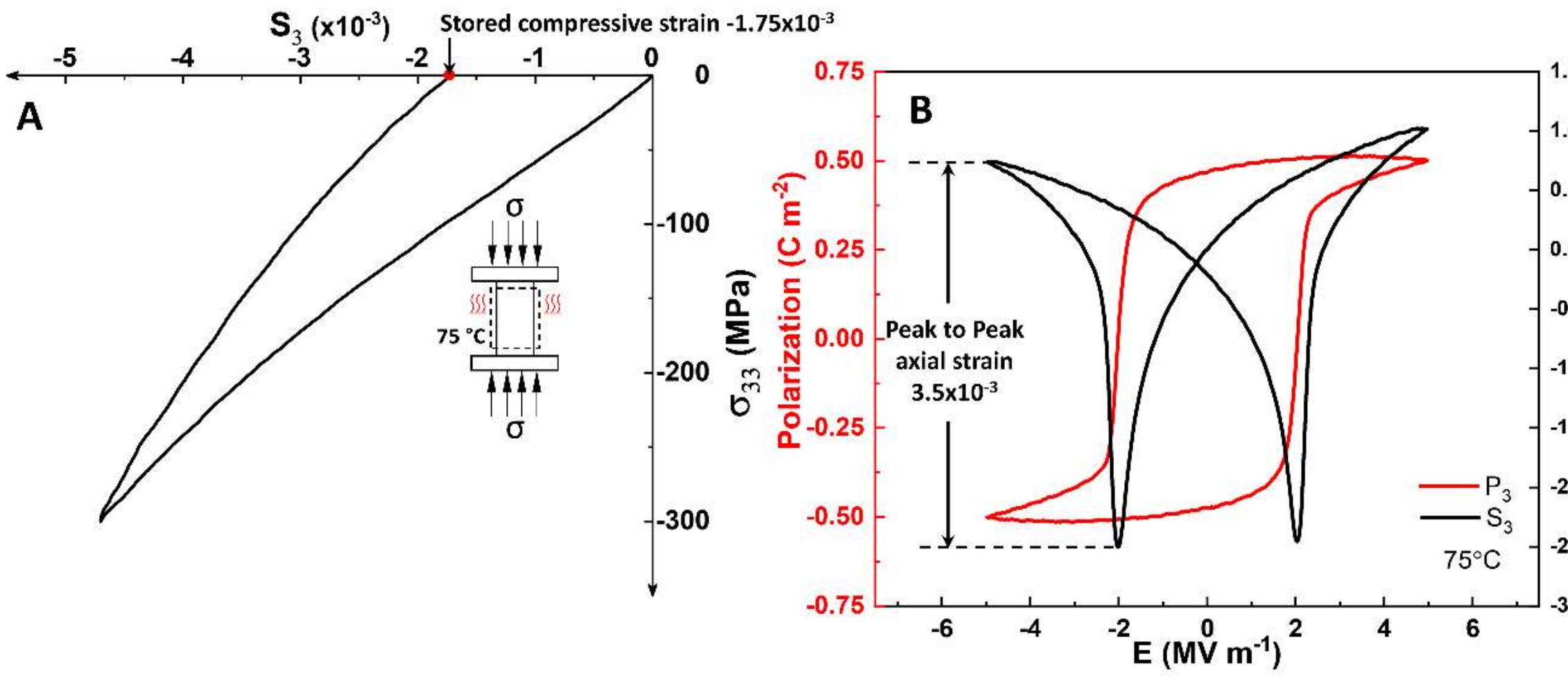

Fig. S14 **Hysteretic mechanical and electric-field-induced strain responses of 0.7BF–0.3BT at 75 °C**. **A**, Uniaxial compressive stress–strain response, showing a stored compressive strain of 1.75×10^{-3} after unloading. **B**, Polarization–electric-field and strain–electric-field hysteresis loops measured at 75 °C, with a peak-to-peak axial strain of 3.5×10^{-3}.

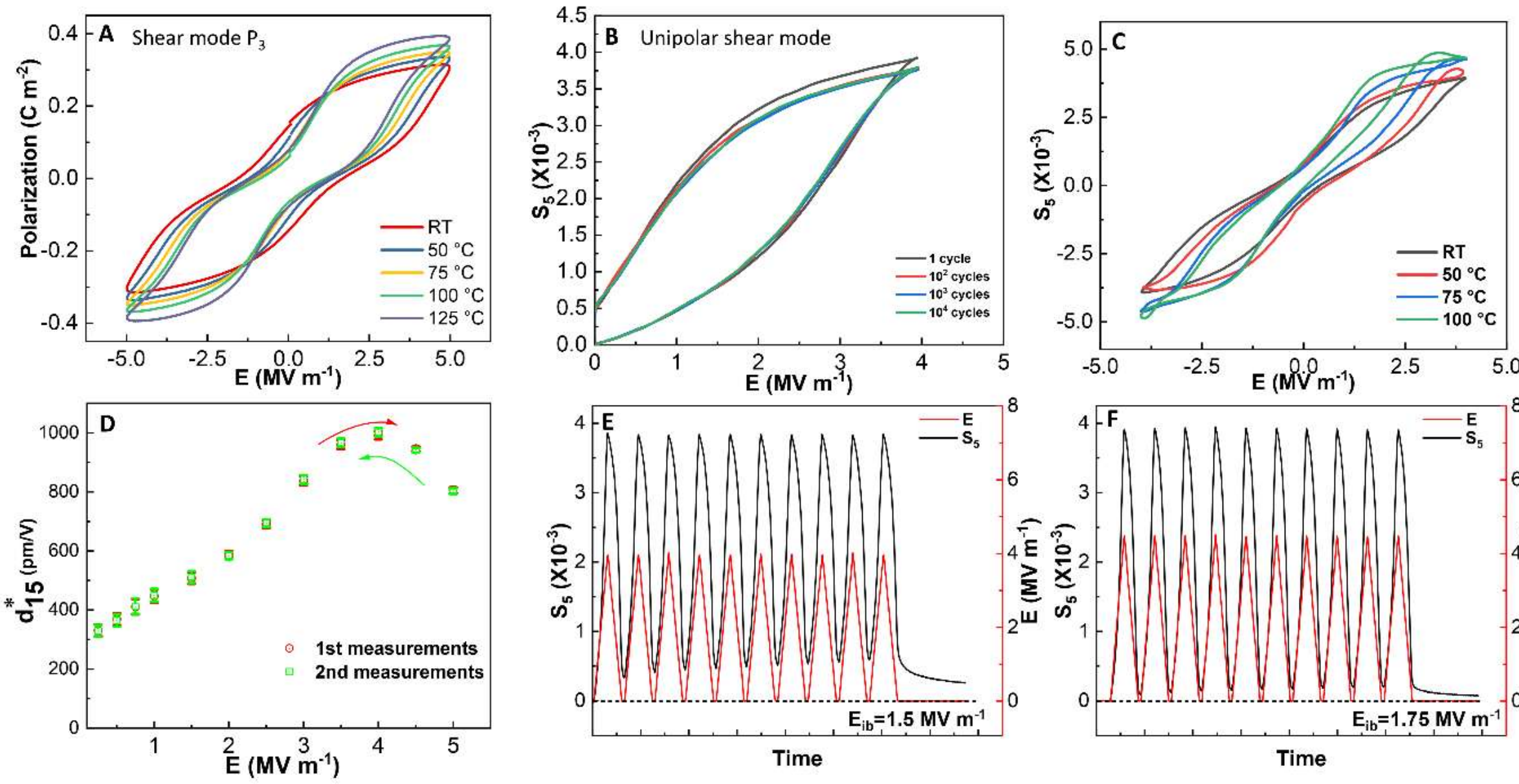

Fig. S15 **Thermal stability and tunability of shear mode electrical domain engineered (E-DE) BFH. A**. temperature dependent axial P_3-E loops at 5 MV m^{-1} measured from RT to 125 °C. **B**. repetition of the unipolar S_5-E measurements at 4 MV m^{-1} for samples cycled under 5 MV m^{-1} for 1, 10^2, 10^2, 10^3 and 10^4 times. **C**. temperature dependent S_5-E loops at 3.75 MV m^{-1} measured from RT to 100 °C. **D**. effective shear piezoelectric coefficient, d_{15}^{*}, of E-DE BFH ceramics measured at various electric field level up to 5 MV m^{-1} and the reversibility of the measurements after exposure to 5 MV m^{-1}. **E** and **F**, the unipolar S_5 responses of E-DE BFH ceramics with E_{ib}=1.5 and 1.75 MV m^{-1} respectively.

Movie. S1 **HAADF-STEM tomography of poled BFH-AQ BF-BT ceramics.**

Movie. S2 **Element partitioning pattern formation process at 550 K of poled BFH-AQ simulated by phase field modelling.**

Movie. S3 **Element partitioning pattern formation process at 550 K and the corresponding evolution of local polarisation of poled BFH-AQ simulated by phase field modelling.**

Movie. S4 **Digital image correlation measurement of shear strain–electric field response at 5 MV m^{-1} at room temperature.**

Movie. S5 **Digital image correlation measurement of shear strain–electric field response at 5 MV m^{-1} at room temperature with the overlay of strain field (S_{31}) mapping.**

Supplementary Text

Phase field modelling

A bulk perovskite single crystal was modelled with a conserved composition field $c(r,t) \in [0.6, 0.8]$ (A-site Bi fraction) and a non-conserved polarisation vector $\mathbf{P} = (P_x + P_y + P_z)$. The total free energy density is:

$$f = f_{Landau}(\mathbf{P}; c, T) + f_{grad}(\nabla\mathbf{P}) + f_{CH}(c, \nabla c) + f_{elastic}(\mathbf{P}, c) + f_{electrostatic}(\mathbf{P}, c, \phi) \qquad \text{(S1)}$$

Where f_{Landau} is a is a composition- and temperature-dependent Landau polynomial for PZT-like endmembers mapped to BF–BT via $x = 1 - c$; f_{grad} is a polarization-gradient energy with G-tensor (G_{11}, G_{44}, G'_{44}); f_{CH} is the Cahn–Hilliard term of the total free energy governs phase separation (spinodal decomposition) and interfacial energy between compositionally distinct regions; $f_{elastic}$ includes periodic microelasticity with Vegard (hydrostatic) and electrostrictive eigenstrains; $f_{ectrostatic}$ is electrostatic energy with short-circuit boundary conditions. The free energy terms are given by:

$$f_{Landau}(\mathbf{P}; c, T) = \alpha_1(P_x^2 + P_y^2 + P_z^2) + \alpha_{11}(P_x^4 + P_y^4 + P_z^4)$$
$$+\alpha_{12}(P_x^2P_y^2 + P_y^2P_z^2 + P_x^2P_z^2) + \alpha_{111}(P_x^6 + P_y^6 + P_z^6)$$
$$+\alpha_{112}[P_x^4(P_y^2 + P_z^2) + P_y^4(P_x^2 + P_z^2) + P_z^4(P_x^2 + P_y^2)] + \alpha_{123}P_x^2P_y^2P_z^2 \qquad \text{(S2)}$$

$$f_{grad}(\nabla\mathbf{P}) = \frac{1}{2}G_{11}(P_{x,x}^2 + P_{y,y}^2 + P_{z,z}^2) + G_{12}(P_{x,x}P_{y,y} + P_{y,y}P_{z,z} + P_{x,x}P_{z,z})$$
$$+\frac{1}{2}G_{44}\left[(P_{x,y} + P_{y,x})^2 + (P_{x,z} + P_{z,x})^2 + (P_{y,z} + P_{z,y})^2\right]$$
$$+\frac{1}{2}G'_{44}[(P_{x,y} - P_{y,x})^2 + (P_{x,z} - P_{z,x})^2 + (P_{y,z} - P_{z,y})^2] \qquad \text{(S3)}$$

where α and G are the Landau coefficients and polarisation gradient energy respectively (*56*,*57*).

$$f_{CH}(c, \nabla c) = \frac{1}{2}A_{chem}(c - c_0)^2 + \frac{1}{4}B_{chem}(c - c_0)^4 + \frac{1}{2}\kappa|\nabla c| \qquad \text{(S4)}$$

where the chemical energy density is described by a fourth polynomial, and κ is the interfacial gradient energy(*58*).

$$f_{elastic}(\mathbf{P}, c) = \frac{1}{2}C_{ijkl}(c)(\varepsilon_{ij} - \varepsilon_{ij}^0)(\varepsilon_{kl} - \varepsilon_{kl}^0) \qquad \text{(S5)}$$

$$\varepsilon_{ij}^0(\mathbf{P}, c) = \alpha_V(c - c_0)\delta_{ij} + \varepsilon_{ij}^Q(\mathbf{P}, c) \qquad \text{(S6)}$$

$$\varepsilon_{xx}^Q = Q_{11}P_x^2 + Q_{12}(P_y^2 + P_z^2) \qquad \text{(S7)}$$

$$\varepsilon_{yy}^Q = Q_{11}P_y^2 + Q_{12}(P_x^2 + P_z^2) \qquad \text{(S8)}$$

$$\varepsilon_{zz}^Q = Q_{11}P_z^2 + Q_{12}(P_x^2 + P_y^2) \qquad \text{(S9)}$$

$$\varepsilon_{xy}^Q = Q_{44}P_x\ P_y\ ,\ \varepsilon_{xz}^Q = Q_{44}P_x\ P_z\ ,\ \varepsilon_{yz}^Q = Q_{44}P_y\ P_z \qquad \text{(S10)-(S12)}$$

where C_{ijkl}, ε_{ij}, ε_{ij}^{0}, α_V and Q are the elastic stiffness , total strain, eigenstrain, Vegard coefficient and electrostrictive coefficients respectively[2].

$$f_{electrostatic}(\mathbf{P}, c, \phi) = \frac{1}{2}\varepsilon_0 K|\mathbf{E}|^2 - \mathbf{E}\cdot\mathbf{P} \text{ with } \mathbf{E} = -\nabla\phi \quad \text{(S13)}$$

Consider the presence of defect with free charge $\rho_{def}(\boldsymbol{r})$ at location $\boldsymbol{r}$:

$$-\varepsilon_0 K\nabla^2\phi = \rho_{def}(\boldsymbol{r}) - \nabla\mathbf{P}(\boldsymbol{r}) \equiv \rho_{total}(\boldsymbol{r}) \quad \text{(S14)}$$

$$\rho_{def}(\boldsymbol{r}) = \rho_{sign}\rho_0(c(r) - c_0) \quad \text{(S15)}$$

where ρ_0 and ρ_{sign} are the charge-scaling constant and sign coefficient respectively (*59*).

All simulations were carried out in a three-dimensional periodic cell of size 256x256x256 grid points. The grid spacing was chosen as $d_x = d_y = d_z = 0.8\ \mathrm{nm}$, giving a simulation box of approximately 205 nm. This high spatial resolution was needed to resolve domain walls and compositional gradients on the order of 1–5 nm. All operators are evaluated spectrally on a periodic 3-D grid using FFTs. The computations were accelerated using GPU-based FFT solvers and an optimized phase-field code (in MATLAB) to handle the large grid. We follow the experimental thermal histories and poling routes used to create BFH microstructures for phase-field modelling, which includes: initialization, pre-poling, zero-field relax, thermal ageing, post ageing relax and property evaluation. The details of each step are given below:

1. Initialization. $c(\mathrm{r}, 0) = c_0 + 0.01\,\eta(\mathrm{r})$ with η zero-mean noise; $\mathrm{P}(\mathrm{r}, 0)$ Gaussian noise (std 0.05 C m^{-2}); Mean composition $c_0 = 0.70$ (i.e., $x = 0.30$).

2. Pre-poling (AQ+Poling analogue). TDGL + electrostatics + elasticity at $T = 300\ \mathrm{K}$ for 500 steps with a dc field $\mathrm{E_{drive}} = 8 \times 10^7\ \mathrm{V\,m^{-1}}$ along $[111]$. Composition is evolved but remains near c_0.

3. Zero-field relax. 300 steps at $E = 0$ to remove transient electrostatic artefacts while retaining the domain texture.

4. Ageing (spinodal decomposition). 20000 steps at $T = 550\ \mathrm{K}$with full CH + TDGL + elasticity + electrostatics; elastic contribution μ_{elastic} is included in μ.

5. Post-ageing relax at room temperature. Additional 500 steps at $E = 0$ to reach a mechanically compatible steady state with frozen-composition at room temperature.

6. Property evaluation (frozen-composition).

Small-signal permittivity vs T: a linearized response was evaluated for $\delta E = 1 \times 10^5\ \mathrm{V\,m^{-1}}$ along $[111]$ from 200–1300 K with TDGL (Time-dependent Ginzburg Landau) relaxation (200 steps) at each T.

Phase field simulation parameters

A_{chem} and B_{chem}: To ensure local coexistence at the ageing temperature T_{age} , we first minimized the Landau energy over $\mathbf{P}$ at fixed c to obtain$f_P^*(c; T_{age})$ and its finite-difference derivative $S(c) =$

$f_P^*/\partial c$. We then chose A_{chem} (with B_{chem} fixed) so that the polarization-renormalized chemical potential $\mu_{local}(c) = A_{chem}(c - c_0) + B_{chem}(c - c_0)^3 + S(c)$ satisfies $\mu_{local}(c_1) = \mu_{local}(c_2)$ for $c_1 = 0.6$ and $c_2 = 0.8$. Then the A_{chem} and B_{chem} can be determined with $f'' > 0$ to enforce the occurrence of spinodal decomposition with targeted stable compositions $c_1 = 0.6$ and $c_2 = 0.8$.

Landau coefficient: Identifying the spinodal compositions in BF-BT theoretically requires knowledge of the free energy as a continuous function of composition. In practice, however, it is very challenging to determine Landau free energy coefficients for the extreme Bi-rich and Ba-rich regions of BF-BT directly. These compositions (near the ends of the solid solution) are inherently unstable or hard to synthesize as single-phase materials – especially the *Bi-enriched and Ba-enriched perovskites with charged A-site segregation*. They exist stably only together (as a coupled two-phase system) because the mixed state allows charge and strain compensation. Obtaining accurate Landau expansion coefficients from experiments or first-principles for such hypothetical "end-member" compositions is not feasible.

To overcome this, two set of Landau coefficients were employed for the phase field simulations and both yielded consistent results with the experiment observations. First, we borrowed Landau-Devonshire parameters from the well-characterized system (PZT) (*60*) on the rhombohedral side and used them as a stand-in for the BF-BT system. Secondly, we established the composition dependent Landau coefficients of $BiFeO_3$-$BaTiO_3$ binary system by a linear interpolation between the two end-members' coefficients of $BiFeO_3$ (*61*) and $BaTiO_3$ (*62*) with a Gaussian type softening at the composition $c = 0.7$.

The composition dependent $C_{ijkl}(c)$ were estimated by mixing the values of end members $BiFeO_3$ and $BaTiO_3$ via Voigt rule with a modest MPB softening at $c = 0.7$. Composition dependent $Q_{11,22,44}(c)$ were also constructed by the same approach using the corresponding values of $BiFeO_3$ and $BaTiO_3$ end members, which employed together with estimated $BiFeO_3$-$BaTiO_3$ landau coefficient for the simulation. The PZT Q coefficient used together with the PZT Landau coefficients for phase field simulation.

The Vegard coefficient ($\alpha_V = -0.028$) was calculated based on the difference between the lattice parameters for the compositions of $c_1 = 0.6$ and $c_2 = 0.8$ (i.e. $Bi_{0.6}Ba_{0.4}Fe_{0.7}Ti_{0.3}$ and $Bi_{0.8}Ba_{0.2}Fe_{0.7}Ti_{0.3}$ respectively). The pseudocubic lattice parameters of each composition were estimated by the averaged lattice parameters determined by the A-O and B-O sublattices. The charge-scaling constant $\rho_0 = eN_s(z_{Bi} - z_{Ba})$, where the density of A-site $N_s = a^{-3} \approx (4.0\ \text{Å})^{-3} = 1.6 \times 10^{28} m^{-3}$, with $z_{Bi} - z_{Ba} = 1$ and $e = 1.602 \times 10^{-1}$ C, $\rho_0 \approx 2.6 \times 10^{9}\ \mathrm{C\ m^{-3}}$.

REFERENCES

1. A. J. Bell, T. P. Comyn, T. J. Stevenson, Expanding the application space for piezoelectric materials. *APL Mater.* **9**, 010901 (2021).

2. L. E. Cross, S. J. Jang, R. E. Newnham, S. Nomura, K. Uchino, Large electrostrictive effects in relaxor ferroelectrics. *Ferroelectrics* **23**, 187–191 (1980).

3. K. Uchino, Electrostrictive and piezoelectric effects in relaxor ferroelectrics: Historical background. *IEEE Trans. Ultrason. Ferroelectr. Freq. Control* **69**, 3013–3036 (2022).

4. R. Ramesh, D. G. Schlom, Creating emergent phenomena in oxide superlattices. *Nat. Rev. Mater.* **4**, 257–268 (2019).

5. P. Yu, W. Luo, D. Yi, J. X. Zhang, M. D. Rossell, C. H. Yang, L. You, G. Singh-Bhalla, S. Y. Yang, Q. He, Q. M. Ramasse, R. Erni, L. W. Martin, Y. H. Chu, S. T. Pantelides, S. J. Pennycook, R. Ramesh, Interface control of bulk ferroelectric polarization. *Proc. Natl. Acad. Sci. U.S.A.* **109**, 9710–9715 (2012).

6. M. Dawber, K. M. Rabe, J. F. Scott, Physics of thin-film ferroelectric oxides. *Rev. Mod. Phys.* **77**, 1083–1130 (2005).

7. D. G. Schlom, L.-Q. Chen, C.-B. Eom, K. M. Rabe, S. K. Streiffer, J.-M. Triscone, Strain tuning of ferroelectric thin films. *Annu. Rev. Mater. Res.* **37**, 589–626 (2007).

8. J. W. Cahn, On spinodal decomposition. *Acta Metall.* **9**, 795–801 (1961).

9. O. V. Boytsova, O. N. Makarevich, D. I. Sharovarov, A. M. Makarevich, Spinodal decomposition in the chemistry and technology of inorganic materials. *Inorg. Mater.* **58**, 673–686 (2022).

10. Y. Li, Z. Yang, Y. Chen, Z. Zhang, Y. Tang, M. Smith, M. Lindley, X. Cao, D. G. Hopkinson, A. J. Bell, Bulk ferroelectric heterostructures for high temperature lead-free piezoelectrics. arXiv:2507.09673 [cond-mat.mtrl-sci] (2025).

11. Y. Li, Z. Yang, Y. Chen, Z. Zhang, Y.-L. Tang, A. K. Kleppe, E. Koemets, X. Cao, S. J. Milne, J. Pan, Bulk ferroelectric heterostructures: Imprinted actuators. arXiv:2509.06177 [cond-mat.mtrl-sci] (2025).

12. D. P. H. Hasselman, Unified theory of thermal shock fracture initiation and crack propagation in brittle ceramics. *J. Am. Ceram. Soc.* **52**, 600–604 (1969).

13. K. Binder, P. Fratzl, "Spinodal decomposition" in *Phase Transformations in Materials* (Wiley-VCH, 2001), pp. 409–480.

14. T. Yang, C. Dai, V. A. Stoica, F. Xue, H. Wang, Y. Ji, V. Gopalan, L.-Q. Chen, Computing diffraction patterns of microstructures from phase-field simulations. *Acta Mater.* **239**, 118258 (2022).

15. F. Findik, Improvements in spinodal alloys from past to present. *Mater. Des.* **42**, 131–146 (2012).

16. H. Tan, J. Verbeeck, A. Abakumov, G. Van Tendeloo, Oxidation state and chemical shift investigation in transition metal oxides by EELS. *Ultramicroscopy* **116**, 24–33 (2012).

17. Z. Hong, L. Q. Chen, Switchable polar spirals in tricolor oxide superlattices. *Acta Mater.* **164**, 493–498 (2019).

18. Y. Liu, Y. L. Zhu, Y. L. Tang, Y. J. Wang, S. Li, S. R. Zhang, M. J. Han, J. Y. Ma, J. Suriyaprakash, X. L. Ma, Controlled growth and atomic-scale mapping of charged heterointerfaces in $PbTiO_3$/$BiFeO_3$ bilayers. *ACS Appl. Mater. Interfaces* **9**, 25578–25586 (2017).

19. C.-L. Jia, S.-B. Mi, K. Urban, I. Vrejoiu, M. Alexe, D. Hesse, Atomic-scale study of electric dipoles near charged and uncharged domain walls in ferroelectric films. *Nat. Mater.* **7**, 57–61 (2008).

20. L. Liu, K. Xu, Q. Li, Y. Huang, L. Shu, Y.-Y.-S. Cheng, S. Zhang, J. Luo, J. Zhu, J.-F. Li, Ultrathin non-Ising charged domain walls confined in $BiFeO_3$ nanocrystals. *Adv. Funct. Mater.* **32**, 2207730 (2022).

21. J. D. Eshelby, R. E. Peierls, The determination of the elastic field of an ellipsoidal inclusion, and related problems. *Proc. R. Soc. Lond. Ser. A. Math. Phys. Sci.* **241**, 376–396 (1997).

22. R. D. Shannon, Revised effective ionic radii and systematic studies of interatomic distances in halides and chalcogenides. *Acta Crystallogr. A* **32**, 751–767 (1976).

23. E. Sawaguchi, Ferroelectricity versus antiferroelectricity in the solid solutions of $PbZrO_3$ and $PbTiO_3$. *J. Phys. Soc. Jpn.* **8**, 615–629 (1953).

24. L. S. Boehmer, R. W. Smith, An ultrasonic instrument for continuous measurement of sodium levels in fast breeder reactors. *IEEE Trans. Nucl. Sci.* **23**, 359–362 (1976).

25. R. Viswanathan, J. F. Henry, J. Tanzosh, G. Stanko, J. Shingledecker, B. Vitalis, R. Purgert, U.S. program on materials technology for ultra-supercritical coal power plants. *J. Mater. Eng. Perform.* **14**, 281–292 (2005).

26. European Commission, HOTSCAN, CORDIS - EU research results, https://cordis.europa.eu/project/id/262574.

27. Piezo Technologies, Materials Chart, https://piezotechnologies.com/materials-chart/.

28. C. Yang, X. Xu, W. Ali, Y. Wang, Y. Wang, Y. Yang, L. Chen, G. Yuan, Piezoelectricity in excess of 800 pC/N over 400 °C in $BiScO_3$–$PbTiO_3$–$CaTiO_3$ ceramics. *ACS Appl. Mater. Interfaces* **13**, 33253–33261 (2021).

29. S. Ikegami, I. Ueda, Piezoelectricity in ceramics of ferroelectric bismuth compound with layer structure. *Jpn. J. Appl. Phys.* **13**, 1572–1577 (1974).

30. X. P. Jiang, X. L. Fu, C. Chen, N. Tu, M. Z. Xu, X. H. Li, H. Shao, Y. J. Chen, High performance Aurivillius type $Na_{0.5}Bi_{4.5}Ti_4O_{15}$ piezoelectric ceramics with neodymium and cerium modification. *J. Adv. Ceram.* **4**, 54–60 (2015).

31. K. Meng, W. Li, X.-G. Tang, Q.-X. Liu, Y.-P. Jiang, A review of a good binary ferroelectric ceramic: $BaTiO_3$–$BiFeO_3$. *ACS Appl. Electron. Mater.* **4**, 2109–2145 (2022).

32. Z. Rao, B. Dutta, F. Körmann, W. Lu, X. Zhou, C. Liu, A. K. da Silva, U. Wiedwald, M. Spasova, M. Farle, D. Ponge, B. Gault, J. Neugebauer, D. Raabe, Z. Li, Beyond solid solution high-entropy alloys: Tailoring magnetic properties via spinodal decomposition. *Adv. Funct. Mater.* **31**, 2007668 (2021).

33. K. J. Choi, M. Biegalski, Y. L. Li, A. Sharan, J. Schubert, R. Uecker, P. Reiche, Y. B. Chen, X. Q. Pan, V. Gopalan, L. Q. Che, D. C. Schlom, C. B. Eom, Enhancement of ferroelectricity in strained $BaTiO_3$ thin films. *Science* **306**, 1005–1009 (2004).

34. F. Xue, L. Liang, Y. Gu, I. Takeuchi, S. V Kalinin, L.-Q. Chen, Composition- and pressure-induced ferroelectric to antiferroelectric phase transitions in Sm-doped $BiFeO_3$ system. *Appl. Phys. Lett.* **106**, 12903 (2015).

35. S. Tian, J. Xin, Y. Cheng, L. Lai, B. Li, Y. Dai, Strong pinning effect on domains in piezoelectrics. *Acta Mater.* **280**, 120344 (2024).

36. A. M. Flynn, S. R. Sanders, Fundamental limits on energy transfer and circuit considerations for piezoelectric transformers. *IEEE Trans. Power Electron.* **17**, 8–14 (2002).

37. Y. Fuda, K. Kumasaka, M. Katsuno, H. S. Hiroyuki Sato, Y. I. Yoshihiro Ino, Piezoelectric transformer for cold cathode fluorescent lamp inverter. *Jpn. J. Appl. Phys.* **36**, 3050 (1997).

38. M. Acosta, N. Novak, V. Rojas, S. Patel, R. Vaish, J. Koruza Jr. Rossetti G. A., J. Rödel, $BaTiO_3$-based piezoelectrics: Fundamentals, current status, and perspectives. *Appl. Phys. Rev.* **4**, 41305 (2017).

39. K. Uchino, Materials issues in design and performance of piezoelectric actuators: An overview. *Acta Mater.* **46**, 3745–3753 (1998).

40. D. A. Hall, L. Daniel, M. Watson, A. Condie, T. P. Comyn, A. K. Kleppe, P. J. Withers, Domain switching and shear-mode piezoelectric response induced by cross-poling in polycrystalline ferroelectrics. *J. Appl. Phys.* **136**, 194101 (2024).

41. T. Granzow, E. Suvaci, H. Kungl, M. J. Hoffmann, Deaging of heat-treated iron-doped lead zirconate titanate ceramics. *Appl. Phys. Lett.* **89**, 262908 (2006).

42. D. A. Hall, P. J. Stevenson, Field-induced destabilisation of hard PZT ceramics. *Ferroelectrics* **187**, 23–37 (1996).

43. J. F. Ihlefeld, B. M. Foley, D. A. Scrymgeour, J. R. Michael, B. B. McKenzie, D. L. Medlin, M. Wallace, S. Trolier-McKinstry, P. E. Hopkins, Room-temperature voltage tunable phonon thermal conductivity via reconfigurable interfaces in ferroelectric thin films. *Nano Lett.* **15**, 1791–1795 (2015).

44. G. Catalan, J. Seidel, R. Ramesh, J. F. Scott, Domain wall nanoelectronics. *Rev. Mod. Phys.* **84**, 119–156 (2012).

45. S. Pandya, G. A. Velarde, R. Gao, A. S. Everhardt, J. D. Wilbur, R. Xu, J. T. Maher, J. C. Agar, C. Dames, L. W. Martin, Understanding the role of ferroelastic domains on the pyroelectric and electrocaloric effects in ferroelectric thin films. *Adv. Mater.* **31**, e1803312 (2019).

46. V. Mueller, Q. M. Zhang, Shear response of lead zirconate titanate piezoceramics. *J. Appl. Phys.* **83**, 3754–3761 (1998).

47. L. Wang, R. Liang, Z. Zhou, M. Li, M. Gu, P. Wang, X. Dong, Electrical conduction mechanisms and effect of atmosphere annealing on the electrical properties of $BiFeO_3$-$BaTiO_3$ ceramics. *J. Eur. Ceram. Soc.* **39**, 4727–4734 (2019).

48. ASTM International, ASTM C1421, Standard Test Methods for Determination of Fracture Toughness of Advanced Ceramics at Ambient Temperature (ASTM International, 2010).

49. S. Sherrit, H. D. Wiederick, B. K. Mukherjee, PdP135. Nun-iterative evaluation of the real and imaginary material constants of piezoelectric resonators. *Ferroelectrics* **134**, 111–119 (1992).

50. M. Schaffer, B. Schaffer, Q. Ramasse, Sample preparation for atomic-resolution STEM at low voltages by FIB. *Ultramicroscopy* **114**, 62–71 (2012).

51. X. Sang, A. A. Oni, J. M. LeBeau, Atom column indexing: Atomic resolution image analysis through a matrix representation. *Micorsc. Microanal.* **20**, 1764–1771 (2014).

52. M. Nord, P. E. Vullum, I. MacLaren, T. Tybell, R. Holmestad, Atomap: A new software tool for the automated analysis of atomic resolution images using two-dimensional Gaussian fitting. *Adv. Struct. Chem. Imaging* **3**, 9 (2017).

53. M. J. Hÿtch, E. Snoeck, R. Kilaas, Quantitative measurement of displacement and strain fields from HREM micrographs. *Ultramicroscopy* **74**, 131–146 (1998).

54. Y. Li, Y. Chen, Z. Zhang, A. Kleppe, D. A. Hall, In-situ XRD study of actuation mechanisms in $BiFeO_3$-$K_{0.5}Bi_{0.5}TiO_3$-$PbTiO_3$ ceramics. *Acta Mater.* **168**, 411–425 (2019).

55. Y. Li, Z. Zhang, Y. Chen, D. A. Hall, Electric field-induced strain in core-shell structured $BiFeO_3$–$K_{0.5}Bi_{0.5}TiO_3$–$PbTiO_3$ ceramics. *Acta Mater.* **160**, 199–210 (2018).

56. A. F. Devonshire, CIX. Theory of barium titanate—Part II. *Lond. Edinb. Dubl. Philos. Mag. J. Sci.* **42**, 1065–1079 (1951).

57. Y. L. Li, S. Y. Hu, Z. K. Liu, L. Q. Chen, Phase-field model of domain structures in ferroelectric thin films. *Appl. Phys. Lett.* **78**, 3878–3880 (2001).

58. J. W. Cahn, J. E. Hilliard, Free energy of a nonuniform system. I. Interfacial free energy. *J. Chem. Phys* **28**, 258–267 (1958).

59. M. Z. Bazant, Theory of chemical kinetics and charge transfer based on nonequilibrium thermodynamics. *Acc. Chem. Res.* **46**, 1144–1160 (2013).

60. M. J. Haun, E. Furman, S. J. Jang, L. E. Cross, Thermodynamic theory of the lead zirconate-titanate solid solution system, Part V: Theoretical calculations. *Ferroelectrics* **99**, 63–86 (1989).

61. L. Li, X. Cheng, J. R. Jokisaari, P. Gao, J. Britson, C. Adamo, C. Heikes, D. G. Schlom, L.-Q. Chen, X. Pan, Defect-induced hedgehog polarization states in multiferroics. *Phys. Rev. Lett.* **120**, 137602 (2018).

62. Y. L. Li, L. E. Cross, L. Q. Chen, A phenomenological thermodynamic potential for $BaTiO_3$ single crystals. *J. Appl. Phys.* **98**, 64101 (2005).
